# Compound danshen dripping pills modulate the perturbed energy metabolism in a rat model of acute myocardial ischemia

**DOI:** 10.1038/srep37919

**Published:** 2016-12-01

**Authors:** Jiahua Guo, Yonghong Yong, Jiye Aa, Bei Cao, Runbin Sun, Xiaoyi Yu, Jingqiu Huang, Na Yang, Lulu Yan, Xinxin Li, Jing Cao, Nan Aa, Zhijian Yang, Xiangqing Kong, Liansheng Wang, Xuanxuan Zhu, Xiaohui Ma, Zhixin Guo, Shuiping Zhou, He Sun, Guangji Wang

**Affiliations:** 1 Key Laboratory of Drug Metabolism and Pharmacokinetics, State Key Laboratory of Natural Medicines, Key laboratory of drug design and optimization, China Pharmaceutical University, No. 24 TongjiaLane, Nanjing, 210009, China; 2 State Key Laboratory of Core Technology in Innovative Chinese Medicine, Tasly R&D Institute, Tianjin Tasly Group Co., Ltd., No. 2 Pujihe East Road, Tianjin, 300410, China; 3 Department of Cardiology, The First Affiliated Hospital of Nanjing Medical University, No. 300 Guangzhou Avenue, Nanjing, 210029, China; 4 Key Lab of Chinese Medicine, Nanjing University of Chinese Medicine, No. 282 Hanzhong Road, Nanjing, 210029, China; 5 School of Pharmaceutical Science and Technology, Tianjin University, No. 92 Weijin Road, Tianjin, 300072, China

## Abstract

The continuous administration of compound danshen dripping pills (CDDP) showed good efficacy in relieving myocardial ischemia clinically. To probe the underlying mechanism, metabolic features were evaluated in a rat model of acute myocardial ischemia induced by isoproterenol (ISO) and administrated with CDDP using a metabolomics platform. Our data revealed that the ISO-induced animal model showed obvious myocardial injury, decreased energy production, and a marked change in metabolomic patterns in plasma and heart tissue. CDDP pretreatment increased energy production, ameliorated biochemical indices, modulated the changes and metabolomic pattern induced by ISO, especially in heart tissue. For the first time, we found that ISO induced myocardial ischemia was accomplished with a reduced fatty acids metabolism and an elevated glycolysis for energy supply upon the ischemic stress; while CDDP pretreatment prevented the tendency induced by ISO and enhanced a metabolic shift towards fatty acids metabolism that conventionally dominates energy supply to cardiac muscle cells. These data suggested that the underlying mechanism of CDDP involved regulating the dominant energy production mode and enhancing a metabolic shift toward fatty acids metabolism in ischemic heart. It was further indicated that CDDP had the potential to prevent myocardial ischemia in clinic.

Cardiovascular diseases (CVDs) are the leading cause of morbidity and mortality in both industrialized and developing countries, accounting for approximately 30% of all deaths^1^. The incidence of ischemic heart disease is increasing^2^,^3^, and stable angina pectoris is one of the most frequent clinical symptoms of CVDs. Stable angina pectoris is associated with the imbalance between myocardial oxygen demand and supply due to transient myocardial ischemia. In general, patients with stable angina pectoris and no obstructive coronary artery disease (CAD) are at high risk for cardiovascular events^4^. Thousands of years of traditional Chinese medicine (TCM) have yielded a modernized pharmaceutical preparation-based TCM therapeutic, compound danshen dripping pills (CDDP, Dantonic^®^, T89, FufangDanshenDiwan in Chinese), which show potential in the management of myocardial ischemia. CDDP consists of *Radix Salvia miltiorrhiza* (Labiatae sp. plant, Danshen in Chinese), *Radix Notoginseng* (Araliaceae plant, Sanqi in Chinese) and *Borneolum* (Bingpian in Chinese)^5^. CDDP has been listed in the Chinese Pharmacopeia since 1990 and has been widely applied for the clinical prevention and treatment of angina in the context of myocardial ischemia and other cardiovascular conditions^6^. In fact, CDDP has been marketed in many countries (e.g., Canada, Singapore, The United Arab Emirates, Korea, Russia, Cuba, Vietnam, India, and South Africa) as a dietary supplementary for the prevention and treatment of ischemic heart diseases^7^. The Phase III clinical trial approved by the US Food and Drug Administration (NCT01659580) in 2013 has been successfully completed by the end of March, 2016.

Previous studies revealed that the pharmacological mechanism of action of CDDP involved modulating platelet and leucocyte function, reducing circulating adhesion molecules, ameliorating myocardial fibrosis, protecting against microcirculatory disturbances, alleviating myocardial damage, and inhibiting NADPH oxidase^8^,^9^,^10^,^11^,^12^,^13^,^14^. These results suggested the ability of CDDP to alleviate the biochemical stress of myocardial ischemia and enhanced our understanding of its therapeutic benefits and underlying mechanisms of action. The data from phase II clinical trial demonstrated the strong efficacy of long-term CDDP treatment in relieving myocardial ischemia. In detail, within the first four weeks of CDDP treatment, the pharmacodynamic (PD, exercise tolerance test, ETT) curve fluctuated in a similarly paralleled way to that of pharmacokinetics (PK) during the short-term CDDP treatment. Interestingly, after administering CDDP over eight weeks, the efficacy became constant in the volunteers, despite of the fluctuation of PK. However, until now, the underlying mechanism of this interesting phenomenon, especially regarding to the impact of CDDP treatment on the metabolism stress and physiological status of ischemic heart tissue, is poorly understood. Fatty acids and glucose are the primary myocardial energy sources^15^,^16^. The oxidation of glucose and fatty acids utilizes different amounts of oxygen, and these processes impose a differential burden on cardiac muscle cells for the sufficient production of ATP^17^,^18^,^19^. Because energy flow and metabolic flow are crucially important for assessing the effects of CDDP on ischemic heart tissue, metabolomic profiling was performed in this study to provide further insight into the efficacy of CDDP.

Clinically, metabolomics/metabonomics has been employed in diagnostics, the identification of biomarkers for various diseases, drug target discovery and the evaluation of drug efficacy^20^,^21^,^22^,^23^. Metabolomic analysis can provide a unique insight into the dynamic and complex metabolic changes in the body in response to a disease or a chemical intervention. Those changes illustrate the metabolic status of small molecules, which is complementary to alterations in protein and RNA levels. To date, few studies have investigated endogenous molecules involved in metabolic and energy flow in both myocardium and the peripheral circulatory system or the dynamic changes in endogenous compounds after CDDP administration. The current study was designed to investigate the metabolic changes that occur in the heart and in the peripheral circulatory system upon acute myocardial ischemia. We analyzed molecules over time in the plasma and heart tissue in the isoproterenol (ISO) induced myocardial ischemia rat model to explore the modulation of metabolic and energy flow by CDDP.

## Results

### Effect of CDDP on HW/tibia length in the ISO-induced model rats

**Figure 1:**
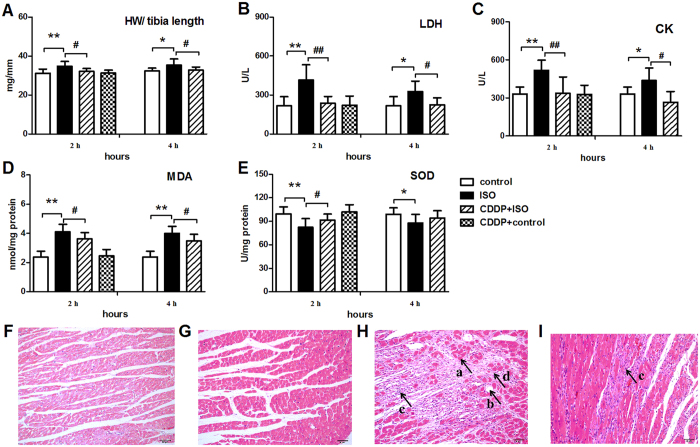
Effect of CDDP on the HW/tibia length index, plasma LDH and CK levels, myocardium MDA level and SOD activity and the myocardial tissue structure in ISO-induced model rats. (**A**) ratio of heart weight and tibia length (HW/tibia length); (**B**) lactate dehydrogenase (LDH); (**C**) creatine kinase (CK); (**D**) malondialdehyde (MDA); (**E**) superoxide dismutase (SOD); (**F**) H & E staining in the control group; (**G**) H & E staining in the CDDP + control group; (**H**) H & E staining in the ISO group; (**I**) H & E staining in the CDDP + ISO group. Data are presented as the mean ± S.D. (n = 10). **P < 0.01 vs control; *P < 0.05 vs control; ^##^P < 0.01 vs ISO model; ^#^P < 0.05 vs ISO model. The black arrows refer to ISO-damaged areas in the left ventricle. a: Myocardial cell necrosis; b: Edema; c: Inflammatory cell infiltration; d: Disrupted myocardial fiber (magnification, X200).

In general, subcutaneous injection of ISO for two days increased the index of heart weight and tibia length ratio (HW/tibia length) compared with the controls, indicating the occurrence of myocardial edema and inflammation after ischemia. The index of HW/tibia length showed no significant difference between CDDP pretreatment in normal control group and normal control group. The CDDP pretreatment significantly decreased the index of HW/tibia length relative to the ISO-induced model rats (Fig. 1A).

### Effects of CDDP on CK and LDH plasma levels in the ISO-induced model rats

As shown in Fig. 1, the LDH and CK levels were significantly increased in the ISO-induced model rats at 2 and 4 h (Fig. 1B,C). There was no significant difference between CDDP pretreatment in normal control group and normal control group (2 h). CDDP (167 mg/kg) daily pretreatment for 10 consecutive days in ISO-induced model group markedly reduced CK and LDH levels relative to the ISO-induced model (2 h, 4 h).

### Effects of CDDP on the myocardium MDA level and SOD activity in the ISO-induced model rats

MDA is the principal and most studied product of polyunsaturated fatty acid peroxidation. As shown in Fig. 1, the content of myocardium MDA increased significantly after introduction of ISO at 2 and 4 h, as compared with the normal control group (Fig. 1D). Pretreatment with CDDP for 10 consecutive days apparently restrained the increase in MDA level evoked by ISO (2 h, 4 h). Activity of the antioxidant enzyme SOD was significantly reduced in the ISO-induced model rats at 2 and 4 h (Fig. 1E). CDDP daily pretreatment for 10 consecutive days in ISO-induced model group markedly increased SOD activity relative to the ISO-induced model (2 h, 4 h). However, there was no significant difference between CDDP pretreatment in normal control group and normal control group on both the MDA level and SOD activity (2 h).

### Effect of CDDP on myocardial histopathology

The histopathological inspection of myocardial tissue showed ISO-induced myocardial injury and necrosis (Fig. 1H). For example, in the left ventricle, obvious myocardial cell necrosis, inflammatory cell infiltration, sub-endocardial hyperemia and edema, and local collagen fiber hyperplasia were observed. No obvious difference was detected between CDDP pretreatment in normal control group and normal control group (Fig. 1F,G). While, pretreatment with CDDP in ISO-induced model group significantly ameliorated the ISO-induced abnormalities in the myocardial tissue; normal cardiac muscle fibers with clear transverse striations, fewer inflammatory cells, and no obvious necrosis or edema were observed (Fig. 1I). The quantification of histological lesions was shown in Supplementary Table S1.

### Effect of CDDP on energy flow in the ISO-induced model rats

**Figure 2:**
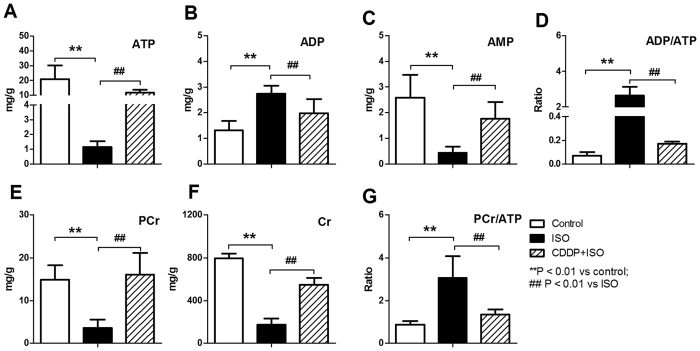
Effect of CDDP on energy flow in ISO-induced model rats after administration of CDDP for 2 h. (**A**) ATP; (**B**) ADP; (**C**) AMP; (**D**) ADP/ATP ratio; (**E**) PCr; (**F**) Cr; (**G**) PCr/ATP ratio. Data are presented as the mean ± S.D. (n = 6). **P < 0.01 vs control; ^##^P < 0.01 vs ISO.

In general, ISO markedly decreased energy generation, i.e., reduced the levels of ATP, AMP, PCr and Cr in heart tissue and increased ADP level and the PCr/ATP and ADP/ATP ratios (Fig. 2). In contrast, pretreatment with CDDP in ISO-induced model group prevented these effects induced by ISO.

### GC/MS profiles of plasma metabolites and overview of the plasma data

**Figure 3:**
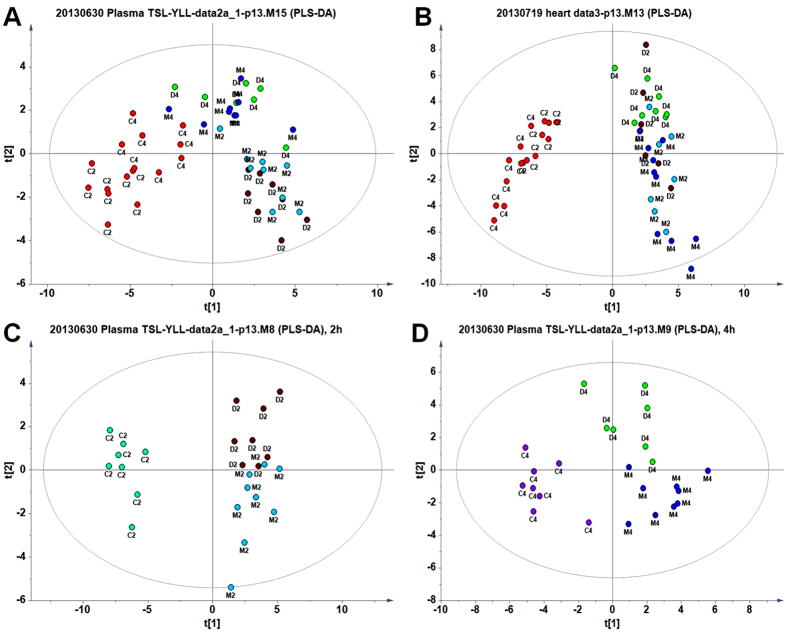
PLS-DA scores plots of the ischemia myocardial model rats and the rats administrated with CDDP for 2 h and 4 h. (**A**) plasma samples, 2 h and 4 h; (**B**) heart tissues, 2 h and 4 h; (**C**) plasma samples, 2 h; (**D**) plasma samples, 4 h. C: control; M: ISO; D: CDDP + ISO; 2: 2 h; 4: 4 h.

Visual inspection of the GC/MS profile of plasma metabolites revealed differences between the model and the control animals (Supplementary Fig. S1). Deconvolution of the chromatograms produced 116 peaks in the plasma extracts, and 64 metabolites were identified, including amino acids, small organic acids, carbohydrates, fatty acids, lipids, and amines (Supplementary Table S2). To acquire the quantitative data, a quant mass was chosen for each molecule, and the peak area was obtained for each deconvoluted peak/molecule. Based on the data matrix (with the two vectors of observations/samples and variables/molecules), an unsupervised PCA model was applied to provide an overview of the data set. No outlier was found in the PCA model (data not shown). A PLS-DA model was then created with the samples classified into five groups: ISO-induced model animals at 2 h and 4 h (M2 and M4), CDDP pretreatment in ISO-induced model animals at 2 h (D2) and 4 h (D4), and normal control animals. The scores plot showed that samples from the same group tended to cluster closely, whereas samples from different groups scattered to different extents (Fig. 3A). In general, induction with ISO for 2 h (M2) and 4 h (M4) caused a distinct shift from control group, whereas administration with CDDP for 2 h (D2) and 4 h (D2) evoked little deviation from the corresponding controls (Fig. 3A,C,D).

A PLS-DA mathematical model was applied to compare the different effects of CDDP on the model and control animals, including normal control (control), CDDP pretreatment in normal control (CDDP + control), ISO-induced model (ISO), CDDP pretreatment in ISO-induced model (CDDP + ISO). The effect of CDDP on the two groups of ISO model and normal control showed a similar trend, although the effective magnitude varied (Supplementary Fig. S2). The slight deviation of CDDP + control group from the normal controls indicated that CDDP had marginal effect on the molecules of plasma in normal controls, at a systematic level. In the general model including more groups of samples, CDDP did not show a significant effect on the regulation of plasma metabolites relative to the ISO-induced model (Fig. 3A); in fact, the CDDP pretreatment in ISO-induced model samples deviated from the ISO-induced model according to the sub-model of the samples collected at 2 and 4 h (Fig. 3C,D). These data showed that administration with CDDP for 2 h affected metabolism and moved the plots slightly towards the controls, although these plots failed to move closer to the left of the controls (Fig. 3C). However, another sub-model of the samples collected at 4 h (Fig. 3D) showed that after administration of CDDP, the plots moved upwards and to the left, closer to the controls on the left than in the model at 2 h (Fig. 3C). These data indicated that administration with CDDP for 4 h had a greater effect on the perturbed metabolism (Fig. 3D).

For the quantitative assessment of the regulatory effect, the relative distance value (RDV) was calculated^24^ based on the PLS-DA sub-model of the plasma data (Fig. 3C,D; Supplementary Table S3). According to the relative distance values, the normalized relative distance between the CDDP + ISO group samples and the ISO group samples increased from 0.322 (2 h) to 0.703 (4 h), while the relative distance between the CDDP + ISO group samples and the control group samples decreased from 3.11 (2 h) to 1.28 (4 h) (Supplementary Table S3), indicating a gradually restoration of the plasma metabolome towards baseline.

### The modulation by CDDP of the plasma metabolites perturbed by ISO

**Figure 4:**
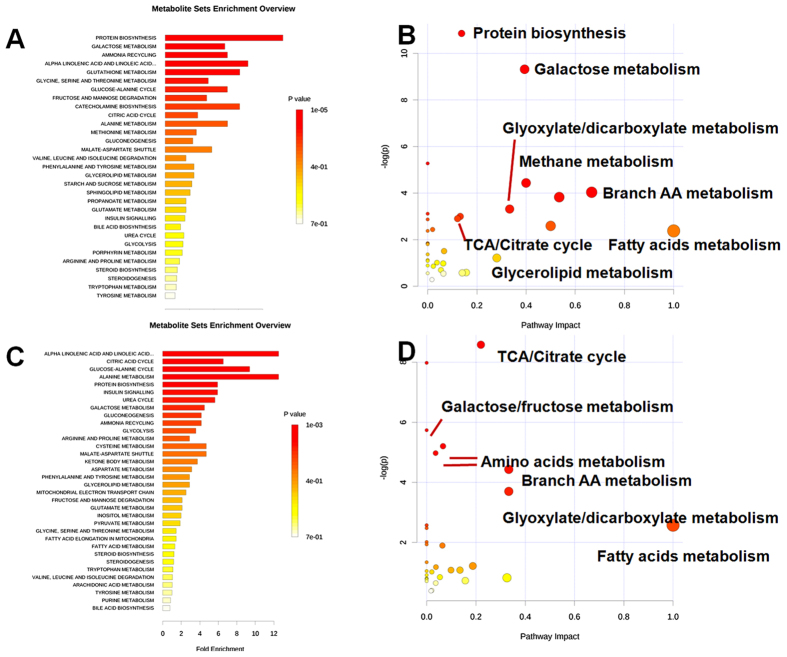
The impact analysis shows the effect of ISO and CDDP on primary metabolic pathways based on the plasma metabolome. (**A**) Enrichment analysis of metabolic sets induced by ISO relative to the normal control; (**B**) Impact analysis of metabolic sets induced by ISO relative to the normal control; (**C**) Enrichment analysis of metabolic sets modulated by CDDP relative to the ISO-induced model; (**D**) Impact analysis of metabolic sets modulated by CDDP relative to the ISO-induced model.

Based on the discriminant metabolites identified in plasma, the metabolic impact analysis revealed that ISO markedly perturbed metabolic pathways involved in amino acid turnover, glycolysis, the TCA cycle, and free fatty acid metabolism (Fig. 4, Supplementary Fig. S3: S-plot). In detail, ISO treatment resulted in a significant reduction in amino acids (glutamine, serine, leucine, valine, tyrosine, glycine, tryptophan, and threonine) and an elevation in carbohydrates (glucose, fructose, galactose, and mannose) (Supplementary Table S4). Pretreatment with CDDP modulated the ISO-induced elevation in carbohydrates and TCA intermediates (citrate, malate, fumarate, and 2-ketoglutarate) at 4 h (Supplementary Table S4; Supplementary Fig. S4). The decreased pyruvate and elevated citrate (2 h) indicated that CDDP enhanced the flux of pyruvate into the TCA cycle (Supplementary Fig. S4B,C), while the marginally decreased glucose (4 h) and increased pyruvate (at 4 h relative to 2 h) and citrate (4 h) suggested enhanced glycolysis (Supplementary Fig. S4A,B,C). ISO induction for a short time (2 h) elevated fatty acids (palmitic acid, linoleic acid, and stearic acid) and their mutual metabolite, 3-hydroxybutyrate (Supplementary Fig. S5; Supplementary Table S4). After further induction of ISO for 4 h, these levels declined to lower than those in the control group. Meanwhile, CDDP significantly increased fatty acids and 3-hydroxybutyrate, indicating that CDDP enhanced the generation and β-oxidation of fatty acids. The up-regulated energy metabolism, including both glycolysis and lipid metabolism, suggested the efficacy of CDDP in relieving the perturbed metabolism induced by acute myocardial ischemia.

### GC/MS profile of metabolites in heart tissue and an overview of the metabolomic data

Visual inspection of the GC/MS profile of metabolites in heart tissue revealed distinct differences between the model animals and the control animals (Supplementary Fig. S6). Deconvolution of the chromatograms resolved 145 peaks in heart tissue, and 75 metabolites were identified, including amino acids, small organic acids, carbohydrates, fatty acids, lipids, amines, and purines (Supplementary Table S5). For the metabolomic study, quantitative data were acquired in the same way as for plasma. Based on the data matrix, an unsupervised PCA model was applied to generate an overview of the data set. No outliers were found in the PCA model. Similar to the plasma results, CDDP showed consistent effect on the two groups of ISO-induced model and normal control (Supplementary Fig. S7). It was indicated that CDDP had a general effect on regulating the metabolome in myocardium regardless of the disease status or not. A PLS-DA model was then created with the samples classified into five groups: ISO-induced model animals for 2 h (M2) and 4 h (M4), CDDP pretreatment in ISO-induced model animals for 2 h (D2) and 4 h (D4), and normal control animals. Similar to the plasma data, the PLS-DA plots in the general model (Fig. 3B) showed that induction with ISO for 2 h (M2) and 4 h (M4) caused the plots to deviate significantly from the normal control plots, indicating that ISO strongly perturbed the metabolites in heart tissue.

Two sub-models of the tissue samples collected at 2 h and 4 h were calculated. The PLS-DA models of 3 principal components and the OPLS models clearly showed that administration with CDDP for 2 h and 4 h abolished the tendency towards deviation induced by ISO, and the CDDP + ISO group samples tended to move closer to control group samples (Supplementary Figs S8, S9), indicating a recovery of the perturbed metabolism towards the normal baseline.

For the quantitative assessment of the regulatory effect, the relative distance value (RDV) was calculated^24^ based on the PLS-DA sub-model of the heart tissue data (Supplementary Figs S8, S9; Supplementary Table S3). According to the relative distance values, the normalized relative distance between the CDDP + ISO group samples and the ISO group samples increased from 0.470 (2 h) to 0.628 (4 h), while the relative distance between the CDDP + ISO group samples and the control samples decreased from 1.96 (2 h) to 1.58 (4 h) (Supplementary Table S3), indicating a gradual restoration towards baseline. These data indicated that longer pretreatment with CDDP has the potential to prevent the ISO-induced metabolic perturbations in heart tissue.

### CDDP modulates the ISO-induced perturbations of tissue metabolites

**Figure 5:**
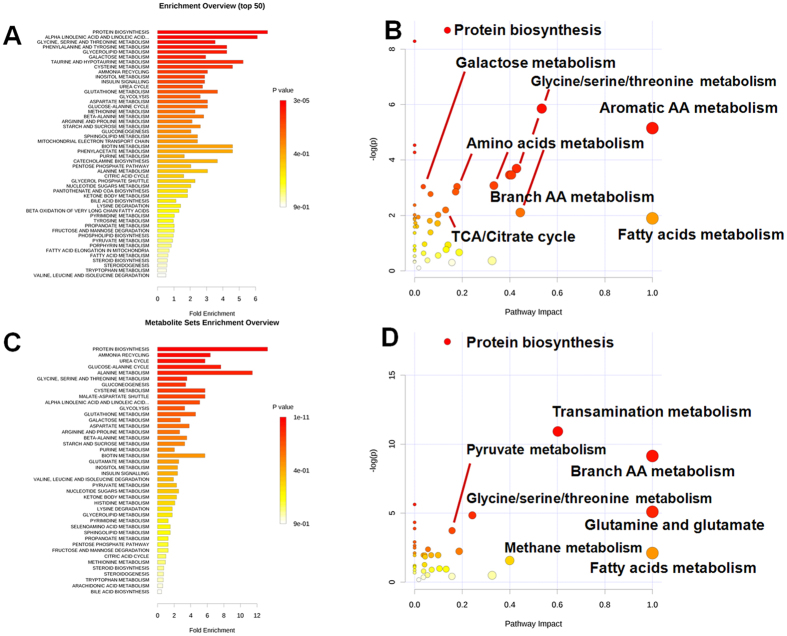
The impact analysis shows the effect of ISO and CDDP on primary metabolic pathways based on the metabolome of heart tissue. (**A**) Enrichment analysis of metabolic sets induced by ISO relative to the normal control; (**B**) Impact analysis of metabolic sets induced by ISO relative to the normal control; (**C**) Enrichment analysis of metabolic sets modulated by CDDP relative to the ISO-induced model; (**D**) Impact analysis of metabolic sets modulated by CDDP relative to the ISO-induced model.

Based on the discriminant metabolites identified in heart tissue, the metabolic impact analysis revealed that ISO induced distinct perturbations in the metabolism of carbohydrates and free fatty acids as well as in the TCA cycle (Fig. 5, Supplementary Fig. S10, S-plot). In detail, ISO induced a significant decrease in carbohydrates (glucose, fructose, galactose, and mannose) and an increase in TCA intermediates (citrate, malate, fumarate and 2-ketoglutarate) and pyruvate (Supplementary Table S6; Supplementary Figs S10, S11), indicating reduced supplementary glucose from the limited blood supply and TCA cycle inhibition due to decreased supplementary oxygen^25^,^26^,^27^. Meanwhile, in the hearts of the model animals, fatty acid levels increased sharply, while the levels of their mutual metabolite, 3-hydroxybutyrate, decreased (4 h), suggesting that fatty acid metabolism was inhibited^28^,^29^. Pretreatment with CDDP in ISO-induced group prevented the elevations in fatty acids and TCA intermediates, indicating an increased supply of glucose and oxygen and thus an enhanced utilization of metabolites for energy supply^11^. The reduction in fatty acids and the increase in their mutual metabolite, 3-hydroxybutyrate, suggested elevated beta-oxidation of fatty acids (Supplementary Fig. S12). The up-regulated energy metabolism (TCA cycle and free fatty acids) suggested the efficacy of CDDP in generating supplementary oxygen and hence alleviating the perturbed metabolism induced by acute myocardial ischemia.

### The regulatory effect of CDDP on the perturbed metabolism in the ISO-induced rat model of acute myocardial ischemia

**Figure 6:**
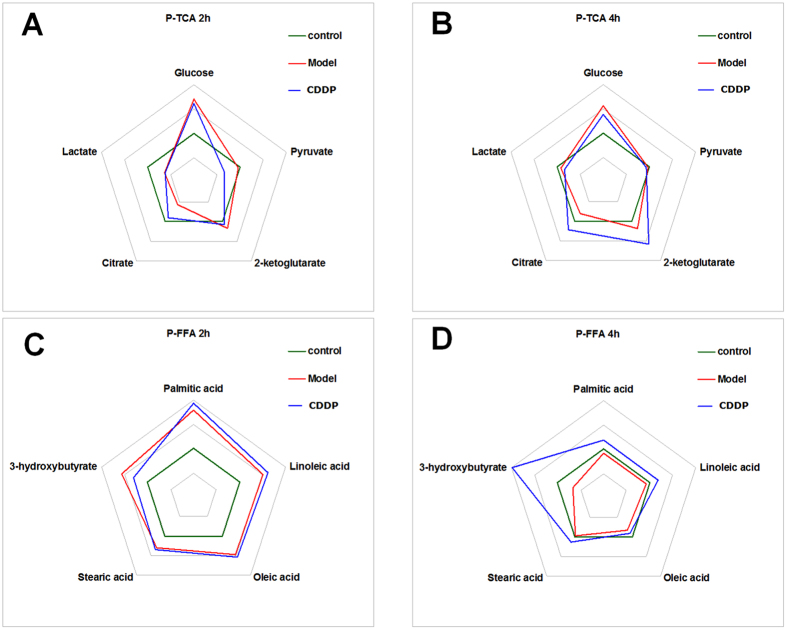
Phase diagram of the 10 key molecules in plasma involved in glycolysis, the TCA cycle and fatty acid metabolism. The phase diagram showed that ISO induced deviations in the TCA cycle and free fatty acid metabolism, especially for the pivotal molecules citrate and 3-hydroxybutyrate, while CDDP pretreatment rectified metabolic homeostasis. (**A**) glycolysis and TCA intermediates, 2 h; (**B**) glycolysis and TCA intermediates, 4 h; (**C**) metabolites involved in fatty acid metabolism, 2 h; (**D**) metabolites involved in fatty acid metabolism, 4 h.

Based on key molecules (5 molecules in the TCA cycle and 5 in fatty acid metabolism) in plasma that were modulated by CDDP, a phase diagram and a radar chart analysis were generated. The phase diagram showed that ISO markedly perturbed the pivotal molecule (citric acid) in the TCA cycle, while CDDP rectified this change such that glycolysis and TCA cycle homeostasis were normalized (Fig. 6A,B). Meanwhile, the phase diagram showed that ISO induced a deviation in free fatty acid metabolism, especially of 3-hydroxybutyrate. CDDP clearly normalized metabolic homeostasis at 4 h (Fig. 6C,D).

**Figure 7:**
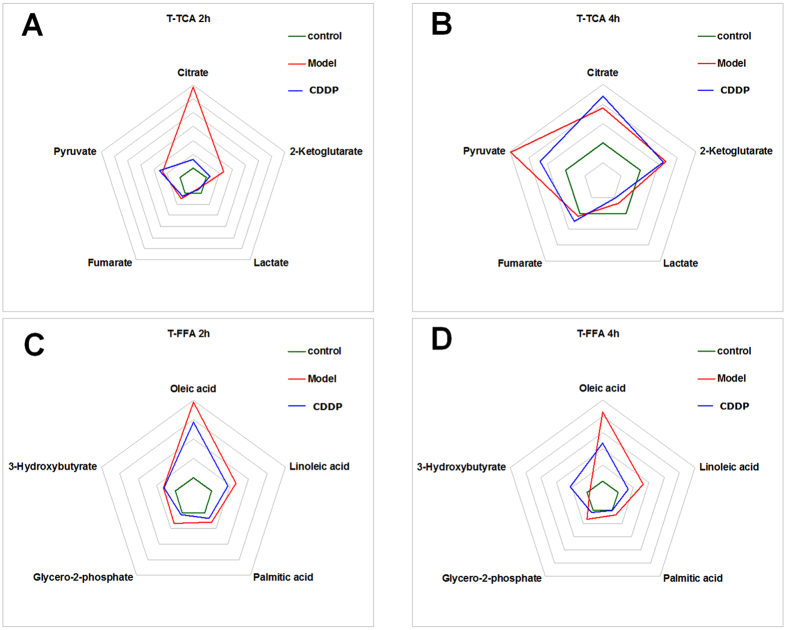
Phase diagram of the 10 key molecules in heart tissue involved in the TCA cycle and fatty acid metabolism. The phase diagram showed that ISO induced obvious deviations in the TCA cycle and in free fatty acid metabolism, especially for the pivotal molecules citric acid, oleic acid and 3-hydroxybutyrate. CDDP pretreatment greatly rectified metabolic homeostasis. (**A**) TCA intermediates, 2 h; (**B**) TCA intermediates, 4 h; (**C**) metabolites involved in fatty acid metabolism, 2 h; (**D**) metabolites involved in fatty acid metabolism, 4 h.

Similarly, based on key molecules (5 molecules in the TCA cycle and 5 in fatty acid metabolism) in heart tissue that were modulated by CDDP, a phase diagram and a radar chart analysis were generated. The phase diagram clearly showed that ISO markedly perturbed the pivotal molecules in the TCA cycle (citric acid, 2-ketoglutarate, fumarate and pyruvate), while CDDP normalized these perturbations (Fig. 7A,B). Meanwhile, the phase diagram showed that ISO induced a significant deviation in free fatty acid metabolism, affecting oleic acid, linoleic acid, palmitic acid, glycerol-2-phosphate, and 3-hydroxybutyrate, while CDDP markedly rectified metabolic homeostasis, especially at 4 h (Fig. 7C,D).

## Discussion

### Perturbation of energy metabolism in myocardial ischemia and the modulatory effects of CDDP

A multi-centered clinical study revealed that continuous administration of CDDP showed good efficacy in relieving myocardial ischemia and alleviating myocardial damage. Previously, plasma and urine metabolomics studies of CDDP revealed that some metabolites involved in energy metabolism, amino acid metabolism, fatty acid metabolism and polyol metabolism changed after ligation of the left ventricular coronary artery for 4 weeks^30^,^31^. Unfortunately, the effects on ischemic heart tissue were not evaluated; furthermore, surgical ligation of the left ventricular coronary artery usually causes breast trauma in model rats and is quite different from clinical transient myocardial ischemia. ISO-induced acute myocardial ischemia is one of the most commonly used models for evaluating the effects of drugs without surgical injury to the animal^26^,^32^,^33^, and the pathophysiological and morphological changes in ISO-induced myocardial ischemia are similar to those observed in humans with myocardial ischemia^34^. To probe the underlying mechanism, systemic metabolism markers in peripheral circulation and local metabolism markers in myocardium were evaluated simultaneously in a rat model of acute myocardial ischemia induced by high dose of ISO. The data revealed that model animals showed decreased ATP production^35^ and distinct deviations in biochemical parameters and tissue histopathology (Figs 1 and 2). Consistent with the reduced ATP levels, ISO down-regulated metabolic pathways primarily involved in energy generation, including glycolysis, the TCA cycle and fatty acid metabolism and amino acid turnover (Supplementary Tables S4, S6). In contrast, CDDP pretreatment increased energy production and ATP generation as well as ameliorated biochemical indices and the histopathology induced by ISO. Consistent with the increased energy and ATP generation, CDDP significantly promoted three metabolic pathways that are primarily involved in energy generation, including fatty acid metabolism, the TCA cycle and glycolysis, indicating a marked improvement in local blood circulation^11^ and therefore the supply of supplementary oxygen, glucose and fatty acids. This result was partially supported by a previous study, although it suggested that CDDP modulated only two metabolic pathways, i.e., glycolysis and the TCA cycle^31^. Interestingly, some metabolites, such as citric acid and glucose, showed the same deviating tendency in the model and the CDDP-treated groups in the two independent studies, but lactic acid did not. The inconsistent results might be attributed to the different models, which were induced by ISO for a short duration (2 days) or by surgical ligation of the left ventricular coronary artery for a much longer time (4 weeks). In addition to conventional indices that suggested the efficacy of CDDP, this study showed that the metabolomic profile potentially indicated the regulatory effect of CDDP on the perturbed metabolism in the model animals. In detail, CDDP moderated the deviations induced by ISO, and the metabolomic pattern in the scores plot was rectified towards the normal controls, particularly for the tissue data (Supplementary Figs S8, S9).

### The metabolic modulation may contribute to the CDDP efficacy

The regulating effect of CDDP on fatty acid metabolism, TCA cycle and glycolysis in myocardial tissue suggested that the metabolic modulation might contributing to the efficacy of CDDP in relieving myocardial ischemia. Similarly, a previous study showed that TG could modulate metabolic perturbation in SHR, and reduce blood pressure significantly^36^. Nevertheless TG did not show any distinct activity of known mechanism underscoring its anti-hypertension effect. After administrating TG for eight weeks, the blood pressure of SHR was markedly decreased. Two weeks after the withdrawal of TG and some frontline anti-hypertensive agents, the systolic blood pressure of SHR in all groups increased rapidly, with the exception of those in the TG-treated groups. Interestingly, except for TG, other anti-hypertensive agents failed to regulate metabolic perturbation in SHR. It was suggested that the normalization of fundamental metabolism contributes to the anti-hypertensive effect of TG. Enlightened by the facts above, we hypothesize that the regulation of perturbed metabolism was involved in the underlying mechanism of both TG’s effect on anti-hypertension and CDDP’s effect on myocardial ischemia. In other words, the regulation of perturbed metabolism is a novel pattern that has the potential to rectify ‘disease’ state into normal state.

It is also important to consider the restorative effect of CDDP on the myocardial ischemia, in order to examine its therapeutic efficacy. However, we only determined the preventative effect of CDDP by pretreatment with CDDP in this study. After inducing the acute myocardial ischemia rat model with ISO, the rat recovered gradually, which resulted in a narrow therapeutic time window for the restorative treatment. Additionally, no significant therapeutic effect was detected when CDDP was applied to the ISO-induced acute myocardial ischemia rat during such a short time period; while the longer use of CDDP is meaningless as the self-recovery occurred in the acute myocardial ischemia rats. Thus, only the pretreatment of CDDP was applied and it showed significant effect on modulating energy metabolism. By using the ‘prevention therapy’, i.e., pretreatment of CDDP, the injury of heart tissue and metabolic perturbation induced by ISO was markedly reduced, indicating the effectiveness of CDDP and its potentially clinical value to prevent high risk population from suffering myocardial ischemia. On the other hand, although the effect of CDDP on each of the specific molecules was complex, a general pattern can be observed that CDDP enhanced the energy metabolism, especially for fatty acids metabolism based on the metabolomic data of heart tissues. CDDP showed consistent effect on metabolome in heart tissues of both the ischemic model and the normal control, although to a less extent in plasma. It was further suggested that CDDP possessed a consistently regulatory activity on energy metabolism, despite of their differential metabolomic patterns.

### Potential markers of myocardial ischemia and their differential changes in peripheral blood and local myocardial tissue

To date, the metabolic effects of myocardial ischemia on the whole body are poorly understood, and the metabolic features of local heart tissue and the systemic phenotype of body fluids have not been associated with each other. Because the human body is a complex biological system, the link between the local tissue microenvironment and the systemic macro-environment is not only of crucial importance for understanding the function and nature of living systems but also of key significance for identifying potential markers of myocardial ischemia in systemic fluids, such as plasma. In fact, in the myocardial ischemia model, metabolites were different in myocardium and plasma compared with the control. The metabolic phenotypes of the scores plots (Fig. 3A,B), the distance values (Supplementary Table S3), and the number of discriminatory metabolites revealed that the differences between the model and control tissue samples were greater than those between the model and control plasma samples. In other words, the metabolic differences in plasma were not as significant as those in tissue. The same phenomena was documented in another study on patients with gastric cancer^37^. Although some metabolites showed the same tendency in tissue and plasma from the model animals (for example, glutamine, leucine, valine, glutamate, lactate, pyruvate and 2-ketoglutarate; Supplementary Tables S4 and S6), some pivotal metabolites showed the reverse tendency after ISO induction. For example, ISO induction elevated plasma glucose but reduced citrate. However, in heart tissue, glucose levels significantly decreased, and citrate increased. In consistent with previous study, our study also indicated that an accelerated glycogenolysis was accompanied by an up-regulation of glucose level in the circulation system in model animals of myocardial ischemia^3^. Meanwhile, the accelerated glycolysis in heart resulted in a marked decrease of glucose. In addition to an elevated citrate^27^, and increased level of pyruvate was also observed after myocardial ischemia, partially because the acceleration of glycolysis and inhibition of TCA cycle. Moreover, after the shorter ISO induction (sampling at 2 h on the 2nd day), the major ketone body or the mutual metabolite of fatty acids, 3-hydroxybutyrate, increased in plasma but declined in heart tissue. It was suggested that, after a sudden ischemic event, the produced 3-hydroxybutyrate in liver was delivered more slowly to ischemic heart and other tissues, and a less portion of it in circulation system could be utilized as supplementary fuel substrate in tissues^38^,^39^. Thereafter, level of 3- hydroxybutyrate declined, probably because of an inhibitory feedback effect of 3-hydroxybutyrate on fatty acids beta-oxidation^34^,^36^,^37^. Interestingly, CDDP greatly enhanced the fatty acid metabolism, where fatty acid levels declined, while 3-hydroxybutyrate levels increased dramatically in heart tissue. These molecules were suggested as potential markers of myocardial ischemia and of CDDP efficacy, which were also suggested as the potential markers for clinic patients^40^,^41^,^42^.

### Metabolic shift between glycolysis/TCA and fatty acid metabolism

Fatty acids and glucose are primary myocardial energy sources^15^,^16^. The oxidation of glucose and fatty acids utilizes different amounts of oxygen, and these processes impose a differential burden on cardiac muscle cells for the sufficient production of ATP^17^,^18^,^19^. Usually, a larger portion (approximately 50–70%) of energy is provided by fatty acid oxidation rather than by glycolysis for cardiac muscle cells^15^. Our data showed that ISO had a differential effect on glycolysis/TCA and fatty acid metabolism as well as on amino acid turnover. In detail, ISO increased free fatty acids and reduced 3-hydroxybutyrate in heart, indicating an inhibition of fatty acid metabolism^28^; the reduced glucose and elevated pyruvate and TCA intermediates suggested a greater utilization of glycolysis to supply energy after ISO treatment^43^,^44^. In other words, in response to myocardial ischemia, cardiac muscle cells tended to utilize more glucose and hence make a metabolic shift from primarily fatty acid metabolism to glycolysis^45^. In contrast, CDDP (may have) accelerated fatty acid oxidation, as evidenced by significantly depleted fatty acids (in plasma and heart tissue) and elevated levels of ketone bodies such as 3-hydroxybutyrate (Supplementary Table S6). Meanwhile, the levels of glucose, pyruvate and TCA intermediates were either decreased or not significantly affected, indicating the down-regulation of glycolysis and the TCA cycle. In summary, this study demonstrated that the acute model of myocardial ischemia was involved in perturbed energy metabolism, which was characterized by decreased fatty acids metabolism and elevated glycolysis. For the first time, we provided evidences that CDDP showed distinct modulating effect on energy metabolism induced by myocardial ischemia, i.e., the up-regulation of fatty acids metabolism, and the down-regulation of glycolysis to some extent. CDDP pretreatment prevented the metabolic perturbations induced by ISO and tended to promote a metabolic shift from glycolysis to fatty acid metabolism. This feature may play an important role in revealing the underlying mechanism of action of CDDP. Studies are necessary on the genes and enzymes involved in the metabolic shift between glycolysis and fatty acid metabolism.

## Conclusion

ISO-induced animal model showed obvious myocardial injury, decreased energy production, and a marked change in metabolomic patterns in plasma and heart tissue. ISO increased the levels of free fatty acids and reduced the level of 3-hydroxybutyrate, and reduced glucose and increased pyruvate and TCA intermediates in heart tissue, suggesting the predominant metabolism shift to glycolysis to supply energy. CDDP pretreatment increased energy production, ameliorated biochemical indices, modulated the changes and metabolomic pattern induced by ISO, especially in tissue. CDDP modulated the perturbed metabolism induced by ISO, and enhanced fatty acid metabolism that conventionally dominates the energy supply to cardiac muscle cells. It was indicated that the underlying mechanism of CDDP involves regulating the dominant energy production mode and inducing a metabolic shift toward fatty acid metabolism in ischemic heart tissue. The regulation of energy metabolism is involved in the underlying mechanism of CDDP effect on myocardial ischemia.

## Methods

### Animals

Healthy male Sprague-Dawley (SD) rats, weighing 189 ± 12 g, were purchased from Vital River Lab Animal Technology Co., Ltd. (Beijing, China). The animals were housed in polypropylene cages (each cage housed a maximum of 3 animals) for one week to enable adaptation to the environment at ambient temperature (25 ± 5 °C) and 45 ± 5% relative humidity with a 12-hour light/dark cycle (lights on from 6:00 a.m. to 6:00 p.m.), a standard diet and free access of water. The animals were fasted for 12 h before the experiment and 4 h during the period of samples collection, but free access to water was still allowed. All animals were handled according to the guidelines of the Tasly Animal Research Committee, and the experimental protocols were approved by the Animal Ethics Committee of Tasly Institute (TSL-IACUC-2013-015).

### Agents and materials

Dantonic^®^ (T89, the capsule of Compound danshen dripping pills, lot number: 20120205) was provided by Tasly Pharmaceutical Co., Ltd. (Tianjin, China), 270 mg per capsule. The principal components in Dantonic^®^ were assayed according to the SOP, with the content of Tanshenol of 10.74 (standard limit ranging from 9.33–12.59) mg/g, poly-phenolic acids (including total content of Protocatechuic aldehyde, Salvianolic acid B, Salvianolic acid A, Rosmarinic acid) of 23.04 (18.85–28.26) mg/g, Ginsenoside Rg1 of 5.85 (5.11–7.70) mg/g, and poly-saponins (including total content of Notoginsenoside R1, Ginsenoside Re, Ginsenoside Rb1) of 13.11 (11.56–17.33) mg/g. The reference standards of adenosine 5′-triphosphate disodium salt hydrate (ATP), adenosine 5′-diphosphate sodium salt (ADP), 5′- monophosphate disodium salt (AMP), creatine (Cr), sodium creatine phosphate dibasic tetrahydrate (PCr), the internal standard N6-(6-aminohexyl) adenosine 2,5-diphosphate lithium salt, and isoprenaline hydrochloride (ISO) were purchased from Sigma-Aldrich (St. Louis, USA). The stable-isotope-labeled internal standard compound (IS) myristic-1,2–^13^C_2_ acid (99 atom%^13^C), methoxyamine hydrochloride (purity 98%), and pyridine (≥99.8% GC) were provided by Sigma-Aldrich (St. Louis, USA). N-Methyl-N-trimethylsilyltrifluoroacetamide and 1% trimethylchlorosilane were purchased from Thermo Scientific (Bellefonte, USA). High-performance liquid chromatography grade methanol and n-heptane were obtained from Merck (Darmstadt, Germany). Purified water was produced using a Milli-Qsystem (Bedford, USA). All other reagents were of analytical grade.

### Animal model and treatments

The animals were randomly divided into four groups: (1) normal control (control); (2) CDDP pretreatment in normal control (CDDP + control); (3) ISO-induced model (ISO); (4) CDDP pretreatment in ISO-induced model (CDDP + ISO). The rats in the CDDP + ISO group received CDDP (167 mg/kg equivalent dose for rats according to the Phase II&III clinical trials) dissolved in distilled water (10 ml/kg) by daily intragastric administration for 10 consecutive days and subcutaneous injections (2 ml/kg) of ISO saline solution (85 mg/kg) at 24 h intervals for 2 days (on the 9^th^ and 10^th^ days) only 2 min after CDDP pretreatment^46^,^47^. The CDDP + control group was given CDDP at 167 mg/kg by intragastric administration for 10 consecutive days and administrated normal saline by subcutaneous injections on the 9^th^ and 10^th^ days. The control group and ISO group rats were given distilled water via an intragastric tube for 10 days and injected subcutaneously with normal saline and ISO saline solution, respectively, on the 9^th^ and 10^th^ days. According to the pilot studies, at the end of the experiment, rats were sacrificed under urethane anesthesia (1.3 g/kg, i.p.) at 2 and 4 h after the second ISO injection (rats of CDDP + control group were sacrificed at 2 h after second saline injection). Blood samples (n = 10) were collected via the retro-orbital plexus in heparinized tubes and centrifuged at 4000 rpm for 10 min at 4.0 °C. Plasma was separated and stored at −70 °C for biochemistry assays and the metabolomic study. Meanwhile, the heart tissues (n = 10) were quickly removed, washed with ice-cold saline, blotted dry with filter paper and stored at −70 °C until analysis; the other samples (n = 10) were placed in 10% formalin solution for pathological analysis.

### Biochemical assay and histological inspection

The ratio of the heart weight (HW) to the tibia length was calculated for each rat. Myocardial-specific enzyme activities, including creatine kinase (CK) and lactate dehydrogenase (LDH), were examined using an automatic biochemistry analyzer (Hitachi 7020, Tokyo, Japan). Fractions heart (0.2 g) were homogenized (1:10, w:v) in 0.05 M sodium phosphate buffer (pH 7.4). The level of malondialdehyde (MDA) and the activity of superoxide dismutase (SOD) were measured directly from the heart homogenate by using commercially available kits (Jiancheng, Nanjing, China). To assess ATP, ADP, AMP, PCr, and Cr, each weighed heart tissue sample (0.5 g) was minced and homogenized with pre-cooled methanol-water (1:1, w:v)^48^. The values were measured, and the histological inspection was performed as provided in the supplementary information (Supplementary Method S1).

### Metabolomics study

For the GC/MS analysis, plasma samples were pretreated, extracted and derivatized as previously reported^49^. Myocardial tissue was pretreated, extracted and derivatized in the same way as hepatic tissue^50^. Endogenous molecules in plasma and tissue were profiled using a Shimadzu GCMS-QP2010 (Shimadzu Corp., Tokyo, Japan) equipped with a RTx-5MS column (30 m × 0.25 mm i.d. fused silica capillary column chemically bonded with a 0.25 μm cross bond of 5% diphenyl/95% dimethyl polysiloxane; Restek Corporation, PA, USA)^51^. The raw GC/MS data were processed, and the metabolites were identified^52^. After identification, one feature ion was selected as the quant mass, and the peak area was acquired for each peak/compound^51^. For additional details, please see Supplementary Method S2–4.

### Multivariate statistical analysis

The relative quantitative data for each peak (peak area) was first normalized against the IS, and then, the data matrix was constructed using the normalized peak areas with the sample names as observations in the first column and the retention times/peaks as the response variables in the first row. The multivariate data were evaluated using SIMCA P13 software (Umetrics, Umeå, Sweden), as reported previously (Supplementary Method S5)^53^. For PCA and PLS-DA, all the data was mean-centered and UV scaled, while for OPLS or OPLS-DA, Pareoto scale was applied. For cross validation, the data was randomly divided into seven groups, with six groups as the basic data to calculate the model and one group for check the prediction capacity to determine the number of principal components, where the number of principal components was determined once the Q^2^Y value decreased continuously. Permutation tests were performed with 100 iterations to validate the model. The results of PCA, PLS-DA and OPLS-DA were displayed as scores plots that visualized the clustering of the samples and indicated the similarity of samples. The closer clustering of the samples represented higher compositional similarity, whereas the further clustering represented diverse metabolomic composition. PLS-DA facilitates the visualization of the dynamic and trajectory movement that reflects time-dependent or treatment dependent tendency, while OPLS-DA facilitates the differentiation of groups and the identification of potential markers. The goodness of fit for a model is evaluated using three quantitative parameters; i.e., R^2^X is the explained variation in X, R^2^Y is the explained variation in Y, and Q^2^Y is the predicted variation in Y. The range of these parameters is between 0 and 1, the closer they approached 1, the better they could predict or explain.

### Biomarker and pathway Analysis

Discriminatory compounds were extracted from loading plots constructed following analysis with PLS-DA, and were chosen based on statistic analysis and their contribution to the variation in the models. Metabolic pathway enrichment and topology analysis were performed by Metaboanalyst (http://www.metaboanalyst.ca). Uploaded the discriminatory compounds and embedded Rattus norvegicus (rat) pathway library for pathway analysis and hypergeometric test. A results report was then presented graphically as well as in a detailed table. Potential biomarkers were identified based on the identified metabolic pathways databases such as KEGG (http://www.genome.jp/kegg/), HMDB (http://www.hmdb.ca/), Lipid Maps (http://www.lipidmaps.org/).

### Statistical Analysis

In our present study, the data were expressed as the mean ± S.D. and were analyzed using a one-way analysis of variance (ANOVA) embedded in SPSS (version 18.0) with a significance level of 0.05 or 0.01. All significance tests were two sided.

## Additional Information

**How to cite this article**: Guo, J. *et al*. Compound danshen dripping pills modulate the perturbed energy metabolism in a rat model of acute myocardial ischemia. *Sci. Rep.* **6**, 37919; doi: 10.1038/srep37919 (2016).

**Publisher's note:** Springer Nature remains neutral with regard to jurisdictional claims in published maps and institutional affiliations.

## Supplementary Material

Supplementary Information
