# Supplementary material for: Compound danshen dripping pills modulate the perturbed energy metabolism in a rat model of acute myocardial ischemia
Source: Sci Rep. 2016 Dec 1;6:37919. doi: 10.1038/srep37919 (PMC5131350; doi:10.1038/srep37919)
Supplement: Supplementary Information [file srep37919-s1.doc]

**Supplementary Information to:**

**Compound danshen dripping pills modulate the perturbed energy metabolism in a rat model of acute myocardial ischemia**

Jiahua Guo^1, 2^, Yonghong Yong^3^, ^‡^, Jiye Aa^1*^, Bei Cao^1^, Runbin Sun^1^, Xiaoyi Yu^1^, Jingqiu Huang^1^, Na Yang^1^, Lulu Yan^2^, Xinxin Li^2^, Jing Cao^2^, Nan Aa^3^, Zhijian Yang^3^, Xiangqing Kong^3^, Liansheng Wang^3^, Xuanxuan Zhu^4^, Xiaohui Ma^2,5^, Zhixin Guo^2^, Shuiping Zhou^2^, He Sun^2,5^, Guangji Wang^1*^

1. Key Laboratory of Drug Metabolism and Pharmacokinetics, State Key Laboratory of Natural Medicines, Key laboratory of drug design and optimization , China Pharmaceutical University, No. 24 Tongjia Lane, Nanjing, 210009, China;

2. Tasly R&D Institute, Tianjin Tasly Group Co., Ltd., No.2 Pujihe East Road, Tianjin, 300410, China;

3. Department of Cardiology, The First Affiliated Hospital of Nanjing Medical University, No. 300 Guangzhou Avenue, Nanjing, 210029, China;

4. Key Lab of Chinese Medicine, Nanjing University of Chinese Medicine, No. 282 Hanzhong Road, Nanjing, 210029, China;

5. School of Pharmaceutical Science and Technology, Tianjin University, No. 92 Weijin Road, Tianjin, 300072, China.

* Correspondence to Jiye Aa (Email: [jiyea@cpu.edu.cn](mailto:jiyea@cpu.edu.cn)) or Guangji Wang (Email: guangjiwang@hotmail.com), Tel.: +862583271081, Fax: +862583271060, Key Laboratory of Drug Metabolism and Pharmacokinetics, China Pharmaceutical University, Nanjing, 210009, China.

‡ Co first author.

**Table of Contents**

[**Supplementary Methods** 3](#__RefHeading___Toc448822205)

[Method S1. 3](#__RefHeading___Toc448822206)

[Method S2. 4](#__RefHeading___Toc448822207)

[Method S3. 5](#__RefHeading___Toc448822208)

[Method S4. 5](#__RefHeading___Toc448822209)

[Method S5. 6](#__RefHeading___Toc448822210)

[**Supplementary Tables** 7](#__RefHeading___Toc448822211)

[Table S1. 7](#__RefHeading___Toc448822212)

[Table S2. 7](#__RefHeading___Toc448822213)

[Table S3. 10](#__RefHeading___Toc448822214)

[Table S4. 11](#__RefHeading___Toc448822215)

[Table S5. 12](#__RefHeading___Toc448822216)

[Table S6. 14](#__RefHeading___Toc448822217)

[**Supplementary Figures** 15](#__RefHeading___Toc448822218)

[Figure S1. 15](#__RefHeading___Toc448822219)

[Figure S2. 16](#__RefHeading___Toc448822220)

[Figure S3. 16](#__RefHeading___Toc448822221)

[Figure S4. 17](#__RefHeading___Toc448822222)

[Figure S5. 18](#__RefHeading___Toc448822223)

[Figure S6. 19](#__RefHeading___Toc448822224)

[Figure S7. 20](#__RefHeading___Toc448822225)

[Figure S8. 20](#__RefHeading___Toc448822226)

[Figure S9. 21](#__RefHeading___Toc448822227)

[Figure S10. 22](#__RefHeading___Toc448822228)

[Figure S11.. 23](#__RefHeading___Toc448822229)

[Figure S12. 24](#__RefHeading___Toc448822230)

# Supplementary Methods

**Method S1. Biochemical assay and Histological inspection**

After centrifugation of the homogenate at 12,500 × g for 15 min at 4 ◦C, the supernatant was collected for LC-MS/MS analysis of ATP, ADP, AMP, Crp, and Cr. The LC-MS/MS system consisted of a Surveyor MS pump (Thermo Finnigan, USA), a Surveyor auto-sampler (Thermo Finnigan, USA) and a Thermo Finnigan TSQ Quantum triple quadrupole mass spectrometer (Thermo Finnigan, USA) equipped with an electrospray ionization (ESI) source. Chromatographic separation was achieved on a Hypersil Gold AQ C_18_ column (150 mm × 4.6 mm, 3.0μm, particle, Thermo, USA) at room temperature. The mobile phase consisted of 2 mmol/L ammonium acetate [aqueous](javascript:void(0);) solution (adjusted with ammonia to pH 10.0) at a flow rate of 0.3 mL/min. The mass spectrometer was carried out in the negative mode with spray voltage of 3000v, capillary temperature of 350 ◦C, sheath gas (nitrogen) of 30 psi, auxiliary gas (nitrogen) of 10 psi and collision gas (argon) pressure of 1.5 mm Torr. The optimized quantification was performed using select reaction monitoring(SRM) of the transitions of m/z 505.99 → 159.06 for ATP (CE: 40 eV), m/z 426.09 → 159.05 for ADP (CE: 40 eV), m/z 345.58 → 78.91 for AMP (CE: 52 eV), m/z 210.02 → 79.27 for CrP (CE: 14 eV), m/z 130.03 →88.06 for C (CE: 13 eV), m/z 525.03 → 233.06 for IS (CE: 43 eV). Data acquisition was operated with Xcalibur® (version 1.3) software (Thermo Finnigan).

Myocardial tissues from all the groups were fixed in 10% formalin solution for 48 h at room temperature. The tissues were dehydrated by sequential washes with 70%, 80%, 90%, and 100% ethanol, embedded in paraffin wax(Leica EG1150, Germany) and cut into 3-5μm slices (Leica RM 2235, Germany). One transversal section from the base area of the left ventricle was rehydrated and stained with hematoxylin and eosin(H&E) and examined by an experienced observer who was blind in the test through light microscopy (Nikon 80i, Japan) at 200 ×magnification.

**Method S2. Sample preparation and derivatization**

In brief, plasma were thawed by incubation at 37°C for 20 min before use, and 120µL of methanol containing internal standard [^13^C_2_]-myristic acid (12.5 µg/mL) was added to 30µL plasma. The mixture was vigorously extracted for 3 min and centrifuged with the SORVALL Biofuge Stratos centrifuge (Sollentum, Germany) at 20000 g for 10 min at 4 °C. An aliquot of 100 μL supernatant was transferred to a GC vial, evaporated to dryness using SPD2010-230 SpeedVac Concentrator (Thermo Savant, Holbrook, USA). Thirty micro-liters of methoxyamine in pyridine (10 mg/mL) were added to the dried residue and vigorously vortex-mixed for 2 min. The methoximation reaction was carried out for 16 h at room temperature, followed by trimethylsilylation for 1 h by adding 30 μL of MSTFA with 1% TMCS as the catalyst. At last, the solution was vortex-mixed again for 30s after the external standard methyl myristate in heptane (30 µg/mL) was added to each GC vial for GC/MS analysis.

Frozen heart tissue samples from rats were ground to a homogenous powder using a liquid nitrogen-chilled mortar and pestle. Approximately 30 mg of each pulverized tissue sample was rapidly transferred to 1.5 mL microcentrifuge tube. 900µL of methanol containing internal standard [^13^C_2_]-myristic acid (12.5 µg/mL) was added to each tube. The mixture was vigorously extracted for 3 min and centrifuged with the SORVALL Biofuge Stratos centrifuge (Sollentum, Germany) at 20000 g for 10 min at 4 °C. An aliquot of 200 μL supernatant was transferred to a GC vial, evaporated to dryness using SPD2010-230 SpeedVac Concentrator (Thermo Savant, Holbrook, USA). Thirty micro-liters of methoxyamine in pyridine (10 mg/mL) were added to the dried residue and vigorously vortex-mixed for 2 min. The methoximation reaction was carried out for 16 h at room temperature, followed by trimethylsilylation for 1 h by adding 30 μL of MSTFA with 1% TMCS as the catalyst. At last, the solution was vortex-mixed again for 30s after the external standard methyl myristate in heptane (30 µg/mL) was added to each GC vial for GC/MS analysis.

**Method S3. GC/MS analysis**

Chromatographic separation of the analytes was achieved with a Shimadzu GCMS-QP2010 (Shimadzu Corp., Tokyo, Japan) equipped with a RTx-5MS column (30 m × 0.25 mm i.d. fused-silica capillary column chemically bonded with a 0.25 μm crossbond, 5% diphenyl/95% dimethyl polysiloxane, Restek Corporation, PA, USA). Helium was used as the carrier gas and a temperature starting at 80 °C for 3 min, and then increased at 20 °C/min to 300 °C, where the temperature was held for another 3 min. The elutes were introduced through transfer line into mass spectrometer, and the molecules were ionized at a current beam of 70eV. The masses were scanned over m/z 50-700 with the detector voltage of -1050 V. To minimize systematic variations, all samples were analyzed at a randomized order, and the quantitative data was normalized by the internal standard.

**Method S4. GC/MS data processing and identification of the metabolites**

In brief, the compounds were identified by automatically comparing the MS spectra, in-source fragments, ion features of each peak in the experimental samples with those of reference standards or those available in libraries, such as mainlib and publib in the National Institute of Standards and Technology (NIST) library 2.0 (2012); Wiley 9 (Wiley-VCH Verlag GmbH & Co. KGaA, Weinheim, Germany); the in-house mass spectra library database established by Umeå Plant Science Center (Umeå University, Sweden); and the Key Laboratory of Drug Metabolism and Pharmacokinetics, China Pharmaceutical University (Nanjing, China)

**Method S5. Multivariate statistical analysis**

In a mathematical model for multivariate statistical analysis, each of the samples represents a plot in a N-dimensional space where N stands for the number of variables. Principal component analysis (PCA), partial least squares projection to latent structures and discriminant analysis (PLS-DA), and orthogonal projection to latent structures (OPLS) were used to process the acquired GC/MS data. The modeling involves a mathematical procedure that transforms a number of detected variables into a smaller number of ‘dummy’ variables called principal components (PCs), i.e., by projecting the plots and reducing dimensions to a few principal components that describe the maximum variation of different groups or samples. Cross-validation with seven cross-validation groups was used throughout to determine the number of principal components, and the number of principal components was determined once the Q^2^Y value decreased continuously. Permutation tests were performed with 100 iterations to validate the model. The result of PCA and PLS-DA was displayed as scores plots that visualized the clustering of the samples and indicated the similarity of samples. The closer clustering of the samples represented higher compositional similarity, whereas the further clustering represented diverse metabonomic composition. PLS-DA facilitates the visualization of the dynamic and trajectory movement that reflects time-dependent or treatment dependent tendency, while OPLS facilitates the differentiation of groups and the identification of potential markers. The goodness of fit for a model is evaluated using three quantitative parameters; i.e., R^2^X is the explained variation in X, R^2^Y is the explained variation in Y, and Q^2^Y is the predicted variation in Y. The range of these parameters is between 0 and 1, the closer they approached 1, the better they could predict or explain.

# Supplementary Tables

**Table S1. The blinded and quantitative analysis of histological lesions**

| Group | Necrosis | Edema | Inflammation | Disrupted myocardial fiber |
| --- | --- | --- | --- | --- |
| ISO | ++ | ++ | +++ | ++ |
| control | - | - | - | - |
| CDDP+ISO | + | + | ++ | + |
| CDDP+ control | - | - | - | - |

-: absence; +: mild; ++: moderate; +++: severe; (n=10)

**Table S2.** The identified endogenous compounds in rat plasma

| Identified compounds | Derivatives | m/z | RT (min) |
| --- | --- | --- | --- |
| Pyruvate | MEOX, TMS | 174 | 5.20 |
| 1,3-Propanediol | 2TMS | 130 | 5.27 |
| Lactate | 2TMS | 219 | 5.34 |
| Glycolate | 2TMS | 147 | 5.49 |
| Alanine | 2TMS | 116 | 5.80 |
| 2-Oxoisovalerate | MEOX1, TMS | 89 | 5.87 |
| Oxalate | 2TMS | 190 | 6.08 |
| 3-Hydroxybutyrate | 2TMS | 233 | 6.36 |
| 2-Hydroxyisovalerate | 2TMS | 219 | 6.43 |
| 3-Amino-isobutyrate | 2TMS | 130 | 6.50 |
| Urea1 | 3TMS | 189 | 6.61 |
| 2-Oxoisocaproate | MEOX1, TMS | 216 | 6.87 |
| Valine | 2TMS | 218 | 6.90 |
| Urea2 | 2TMS | 189 | 7.05 |
| Glycerol | 3TMS | 299 | 7.39 |
| Phosphate | 3TMS | 314 | 7.41 |
| Isoleucine | 2TMS | 218 | 7.55 |
| Proline | 2TMS | 142 | 7.60 |
| Glycine | 3TMS | 248 | 7.67 |
| Glycerate | 3TMS | 392 | 7.84 |
| Fumarate | 2TMS | 245 | 7.90 |
| Dihydroxybutyrate | 3TMS | 292 | 7.96 |
| Serine | 3TMS | 218 | 8.06 |
| Acetylglycine | 2TMS | 246 | 8.09 |
| Threonine | 3TMS | 218 | 8.27 |
| Glycine | 2TMS | 248 | 8.70 |
| Aminomalonate | 3TMS | 320 | 8.84 |
| Aminosuccinate | 2TMS | 232 | 8.87 |
| Malate | 3TMS | 233 | 8.94 |
| Adipate | 2TMS | 275 | 9.02 |
| Methionine | 3TMS | 176 | 9.16 |
| Pyroglutamate | 2TMS | 156 | 9.20 |
| Threonate | 4TMS | 292 | 9.34 |
| Creatinine | 3TMS | 329 | 9.44 |
| 2-Ketoglutarate | MEOX, 2TMS | 198 | 9.48 |
| Ornithine | 3TMS | 348 | 9.73 |
| Glutamate | 3TMS | 246 | 9.76 |
| Phenylalanine | 2TMS | 218 | 9.86 |
| Ribose | MEOX, 4TMS | 307 | 10.17 |
| Myristic acid | TMS | 242 | 10.33 |
| Glutamine1 | 4TMS | 317 | 10.45 |
| Glycerol-3-phosphate | 4TMS | 445 | 10.62 |
| Glutamine2 | 3TMS | 245 | 10.66 |
| Citrate | 4TMS | 273 | 10.93 |
| Fructose | MEOX2, 5TMS | 307 | 11.27 |
| Glucose | MEOX2, 5TMS | 319 | 11.35 |
| Glucopyranose | MEOX, 5TMS | 204 | 11.37 |
| Lysine | 4TMS | 317 | 11.47 |
| Mannose | 5TMS | 319 | 11.53 |
| Tyrosine | 3TMS | 218 | 11.57 |
| Mannonate-4-lactone | 4TMS | 217 | 11.69 |
| Gluconic acid, lactone | 4TMS | 217 | 11.77 |
| 2-Keto-gluconate | 5TMS | 204 | 11.82 |
| Palmitoleic acid | TMS | 311 | 11.91 |
| Palmitic acid | TMS | 313 | 12.00 |
| Urate | 4TMS | 456 | 12.37 |
| Myo-Inositol | 6TMS | 305 | 12.39 |
| Linoleic acid | TMS | 337 | 12.79 |
| Oleic acid | TMS | 339 | 12.84 |
| Stearic acid | TMS | 341 | 12.91 |
| Tryptophan | 3TMS | 202 | 12.95 |
| Arachidonic acid | TMS | 117 | 13.50 |
| Cis-4,7,10,13,16,19-Docosahexaenoic acid | TMS | 117 | 14.29 |
| 1-Monopalmitin | 2TMS | 371 | 14.36 |
| 1-Monoolein | 2TMS | 397 | 15.16 |
| 2-Tocopherol | TMS | 502 | 17.99 |
| Cholesterol | TMS | 458 | 18.30 |

**Table S3.** Assessment of the regulatory effect of CDDP based on the relative distance values

| Samples | Types of | Distance values | | | | Reference |
| --- | --- | --- | --- | --- | --- | --- |
| /time | distance values | ISO  to control | CDDP+ISO  to control (M) | CDDP+ISO  to ISO (N) | M/N | figures/models |
| Plasma/ 2 h | Apparent distance values | 10.10 | 10.12 | 3.25 | 3.11 | Figure 3C |
|  | Relative distance values* | 1.000 | 1.002 | 0.322 |  |  |
| plasma/ 4 h | Apparent distance values | 7.11 | 6.41 | 5.00 | 1.28 | Figure 3D |
|  | Relative distance values* | 1.000 | 0.901 | 0.703 |  |  |
| Heart tissue/ 2 h | Apparent distance values | 10.97 | 10.01 | 5.16 | 1.96 | Figure S-8A |
|  | Relative distance values* | 1.000 | 0.913 | 0.470 |  |  |
| Heart tissue/ 4 h | Apparent distance values | 11.05 | 10.98 | 6.94 | 1.58 | Figure S-9A |
|  | Relative distance values* | 1.000 | 0.994 | 0.628 |  |  |

*, the normalized relative distance value was calculated by setting the value between the model and the control as 1.

**Table S4.** The perturbed plasma metabolites induced by ISO and the regulatory effect of CDDP on the metabolites

| Metabolic pathways/metabolites | The regulatory tendency and statistic analysis relative to the controls | | | | | | | | | |
| --- | --- | --- | --- | --- | --- | --- | --- | --- | --- | --- |
| Groups (time) | CDDP+ control | | ISO (2h) | | CDDP +ISO (2h) | | ISO (4h) | | CDDP+ISO (4h) | |
| The controls | control | | control | | ISO | | control | | ISO | |
| Amino acids metabolism |  |  |  |  |  |  |  |  |  |  |
| glutamine, glycine | ± | ／ | ↓↓ | * | ± | ／ | ↓↓ | * | ↑ | ／ |
| serine, leucine, valine, tyrosine, tryptophan, threonine | ↑ | * | ↓↓ | * | ± | ／ | ↓↓ | * | ↑ | ／ |
| Fatty acids |  |  |  |  |  |  |  |  |  |  |
| palmitic acid, linoleic acid, stearic acid | ± | ／ | ↑ | * | ↑ | ／ | ↓ | ／ | ↑ | * |
| 3-hydroxybutyrate | ↓↓ | ** | ↑ | * | ± | ／ | ↓ | * | ↑ | * |
| Carbohydrates and glycolysis |  |  |  |  |  |  |  |  |  |  |
| glucose | ↓↓ | * | ↑ | * | ± | ／ | ↑ | * | ↓ | ／ |
| fructose, galactose, mannose | ± | ／ | ↑ | * | ± | ／ | ↑ | * | ↓ | ／ |
| lactate | ↑↑ | * | ↓ | * | ± | ／ | ± | ／ | ± | ／ |
| pyruvate | ↑ | * | ↑ | ／ | ↓↓ | * | ± | ／ | ± | ／ |
| TCAs |  |  |  |  |  |  |  |  |  |  |
| citrate | ± | ／ | ↓ | * | ↑ | * | ↓ | ／ | ↑ | ／ |
| malate | ↑ | ／ | ↓ | ／ | ± | ／ | ± | ／ | ↑ | ／ |
| fumarate | ± | ／ | ↑ | * | ↑ | ／ | ± | ／ | ↑↑ | ／ |
| 2-keto-glutarate | ↑ | ／ | ↑ | * | ± | ／ | ↑ | * | ↑↑ | ／ |
| glutamate | ↑ | ／ | ↓ | ／ | ↑ | ／ | ↓ | ／ | ↑↑ | ／ |
| glutamine | ± | ／ | ↓ | ／ | ↓ | ／ | ↓ | ／ | ↓ | ／ |

↓, ↓↓: Down-regulated by at least 20% or 50%, respectively; ↑, ↑↑: Up-regulated by at least 20% or 50%, respectively; ±: marginally regulated without statistic significance. *: p<0.05 (one way ANOVA). ／: p>0.05 (one way ANOVA).

CDDP+ control vs control, ISO vs control, and CDDP+ISO vs ISO, respectively

**Table S5. The identified endogenous compounds in heart tissue**

| ID | TMS | m/z | RT (min) |
| --- | --- | --- | --- |
| Pyruvate | MEOX, TMS | 174 | 4.95 |
| Lactate | 2TMS | 191 | 5.10 |
| Alanine | 2TMS | 190 | 5.55 |
| Glycine | 3TMS | 204 | 5.72 |
| Ethanedioate | 2TMS | 190 | 5.84 |
| 3-Hydroxybutyrate | 2TMS | 233 | 6.13 |
| Monomethylphosphate | 2TMS | 241 | 6.31 |
| Urea1 | 3TMS | 261 | 6.37 |
| Valine | 2TMS | 144 | 6.67 |
| Urea2 | 2TMS | 189 | 6.81 |
| 2-Aminoethanol | 2TMS | 174 | 7.10 |
| Isoleucine | 2TMS | 158 | 7.14 |
| Phosphate | 3TMS | 314 | 7.16 |
| Leucine | 2TMS | 158 | 7.32 |
| Proline | 2TMS | 142 | 7.36 |
| Glycine | 3TMS | 248 | 7.43 |
| Glycerate | 3TMS | 292 | 7.61 |
| Uracil | 2TMS | 256 | 7.67 |
| Fumarate | 2TMS | 245 | 7.68 |
| Serine | 3TMS | 218 | 7.83 |
| Threonine | 3TMS | 218 | 8.03 |
| Aspartate | 3TMS | 160 | 8.26 |
| Decanedioic acid | 2TMS | 373 | 8.26 |
| Beta-Alanine | 2TMS | 248 | 8.30 |
| 2,5-Diaminovalerolactam | 2TMS | 160 | 8.52 |
| Aminomalonate | 3TMS | 218 | 8.61 |
| Malate | 3TMS | 233 | 8.71 |
| Aspartate | 2TMS | 100 | 8.92 |
| Pyroglutamate | 2TMS | 230 | 8.96 |
| 4-Aminobutyrate | 3TMS | 304 | 9.00 |
| 6-Azathymine | 2TMS | 256 | 9.05 |
| Cysteine | 3TMS | 220 | 9.16 |
| Creatinine Enol | 3TMS | 329 | 9.20 |
| Glutamate2 | 3TMS | 348 | 9.52 |
| Phenylalanine | 2TMS | 218 | 9.62 |
| N-Acetylaspartate | 3TMS | 158 | 9.84 |
| Taurine | 2TMS | 326 | 9.89 |
| Ribose | MEOX, 4TMS | 217 | 9.94 |
| Glycero-3-phosphate | 4TMS | 243 | 10.19 |
| Glutamine1 | 4TMS | 227 | 10.22 |
| Glycerol-2-phosphate | 4TMS | 299 | 10.39 |
| Glutamine2 | 3TMS | 156 | 10.42 |
| O-Phosphorylethanolamine | 4TMS | 299 | 10.52 |
| Hypoxanthine | 2TMS | 265 | 10.63 |
| Citrate | 4TMS | 273 | 10.70 |
| 2-Ketoglutarate | MEOX, 2TMS | 173 | 10.91 |
| Fructose | MEOX2, 5TMS | 307 | 11.04 |
| Glucose | MEOX2, 5TMS | 319 | 11.12 |
| Lysine | 4TMS | 156 | 11.22 |
| Pentadecanoate | TMS | 299 | 11.28 |
| Tyrosine | 3TMS | 218 | 11.32 |
| Sebacic acid | 2TMS | 373 | 11.47 |
| 1-Decanol | TMS | 215 | 11.51 |
| Octanoic acid | TMS | 201 | 11.60 |
| Palmitoleic acid | TMS | 311 | 11.65 |
| Xanthine | 3TMS | 353 | 11.73 |
| Urate | 4TMS | 441 | 12.13 |
| Palmitic acid | 4TMS | 313 | 12.14 |
| Inositol | 6TMS | 265 | 12.16 |
| Heptadecanoic acid | TMS | 327 | 12.22 |
| Linoleic acid | TMS | 337 | 12.55 |
| Oleic acid | TMS | 339 | 12.57 |
| Stearic acid | TMS | 341 | 12.67 |
| Tryptophan | 3TMS | 202 | 12.71 |
| Undecanedioic acid | 2TMS | 387 | 13.08 |
| Glucose-6-Phosphate | 6TMS | 387 | 13.14 |
| Arachidonic acid | TMS | 117 | 13.26 |
| Cis-5,8,11-Eicosatrienoic acid | TMS | 363 | 13.33 |
| 11,14-Eicosadienoic acid | TMS | 365 | 13.41 |
| 11-Eicosaenoic acid | TMS | 367 | 13.45 |
| 1-Monolinolein | 2TMS | 221 | 13.60 |
| Myo-Inositol-2-Phosphate | 7TMS | 318 | 13.62 |
| 9,12-Octadecadienoic acid | TMS | 408 | 13.68 |
| Cis-4,7,10,13,16,19-Docosahexaenoic acid | TMS | 133 | 14.05 |
| Inosine | 4TMS | 245 | 14.16 |
| 1-Monooleoylglycerol | 2TMS | 397 | 14.86 |
| 1-Monostearin | 2TMS | 399 | 14.95 |
| Turanose | 8TMS | 361 | 15.06 |
| Maltose | 8TMS | 369 | 15.41 |
| Cholesterol | TMS | 368 | 17.70 |

**Table S6.** The perturbed metabolites in heart tissue induced by ISO and the regulatory effect of CDDP on the metabolites

| Metabolic pathways/metabolites | The regulatory tendency and statistic analysis relative to the controls | | | | | | | | | |
| --- | --- | --- | --- | --- | --- | --- | --- | --- | --- | --- |
| Groups (time) | CDDP + control | | ISO ( 2h ) | | CDDP +ISO (2h) | | ISO ( 4h ) | | CDDP+ISO (4h) | |
| The controls | control | | control | | ISO | | control | | ISO | |
| Amino acids metabolism |  |  |  |  |  |  |  |  |  |  |
| glutamine, leucine, valine, tyrosine, tryptophan, threonine | ↑↑ | ** | ↓↓ | * | ± | ／ | ↓↓ | * | ↑ | ／ |
| serine, proline, cysteine, aspartate, alanine, glycine | ↑ | * | ↑ | * | ± | ／ | ± | ／ | ± | ／ |
| Fatty acids metabolism |  |  |  |  |  |  |  |  |  |  |
| palmitic acid, Oleic acid,  linoleic acid, stearic acid | ↑ | * | ↑↑ | * | ↓ | ／ | ↑ | * | ↓ | * |
| 3-hydroxybutyrate | ± | ／ | ↓ | * | ± | ／ | ↓ | * | ↑ | * |
| Carbohydrates and glycolysis |  |  |  |  |  |  |  |  |  |  |
| glucose, fructose, galactose, mannose | ↑ | * | ↓↓ | * | ± | ／ | ↓↓ | * | ↓ | * |
| lactate | ± | ／ | ↓ | * | ± | ／ | ↓ | * | ± | ／ |
| pyruvate | ↑ | ** | ↑↑ | * | ± | ／ | ↑ | * | ↓ | * |
| TCAs |  |  |  |  |  |  |  |  |  |  |
| citrate | ↓↓ | ** | ↑↑ | ／ | ↓↓ | ／ | ↑↑ | ／ | ± | ／ |
| malate | ↓ | ／ | ↑↑ | ／ | ↓ | ／ | ± | ／ | ± | ／ |
| Fumarate | ± | ／ | ↑ | * | ↓ | ／ | ± | ／ | ± | ／ |
| 2-keto-glutarate | ± | ／ | ↑↑ | ／ | ↓↓ | ／ | ↑ | ／ | ↓ | ／ |
| (glutamate) | ± | ／ | ↓ | ／ | ↓ | * | ↓ | ／ | ↓ | * |
| (glutamine) | ↑↑ | ** | ↓↓ | * | ± | ／ | ↓↓ | * | ↓ | * |
| Purine and nucleic acid metabolism |  |  |  |  |  |  |  |  |  |  |
| inosine, xanthine | ± | ／ | ↓↓ | * | ↓ | * | ↓↓ | * | ↓ | ／ |
| uracil | ± | ／ | ↑ | ／ | ↑ | ／ | ↑ | * | ↓ | ／ |

↓, ↓↓: Downregulated by at least 20% or 50%, respectively; ↑, ↑↑: Upregulated by at least 20% or 50%, respectively; ±: marginally regulated without statistic significance. *: p<0.05 (one way ANOVA). ／: p>0.05 (one way ANOVA).

CDDP+ control vs control, ISO vs control, and CDDP+ ISO vs ISO, respectively.

# Supplementary Figures


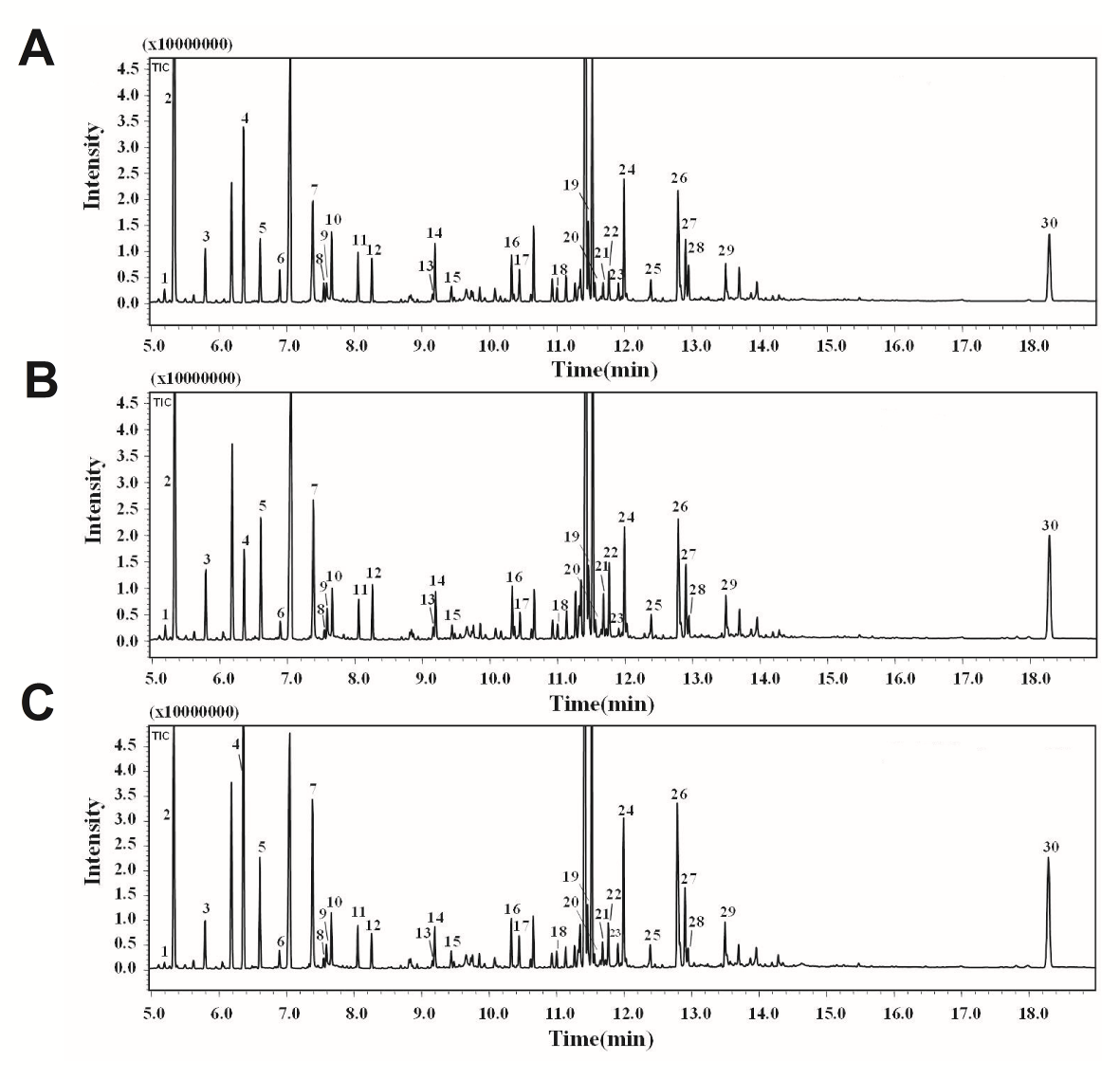


**Figure S1. Typical GC/MS chromatograms of plasma extracts. Visual inspection of the chromatograms revealed obvious differences between the control, ISO-induced model and CDDP pretreatment in ISO-induced model.** A, control; B, ISO; C, CDDP + ISO. The compounds were identified as: 1, pyruvate; 2, lactate; 3, alanine; 4, 3-hydroxybutyrate ; 5, urea; 6, valine; 7, 3 peaks(leucine, glycerol, phosphate) ; 8, isoleucine; 9, proline; 10, glycine; 11, serine; 12, threonine; 13, methionine; 14, pyroglutamate; 15, creatinine; 16, myristic acid; 17, glutamine; 18, citrate; 19, lysine; 20, tyrosine; 21, mannonate-4-lactone; 22, gluconic acid-1,4-lactone; 23, palmitoleic acid; 24, palmitic acid; 25, myo-inositol; 26, linoleic acid; 27, stearic acid; 28, tryptophan; 29, arachidonic acid; 30, cholesterol.


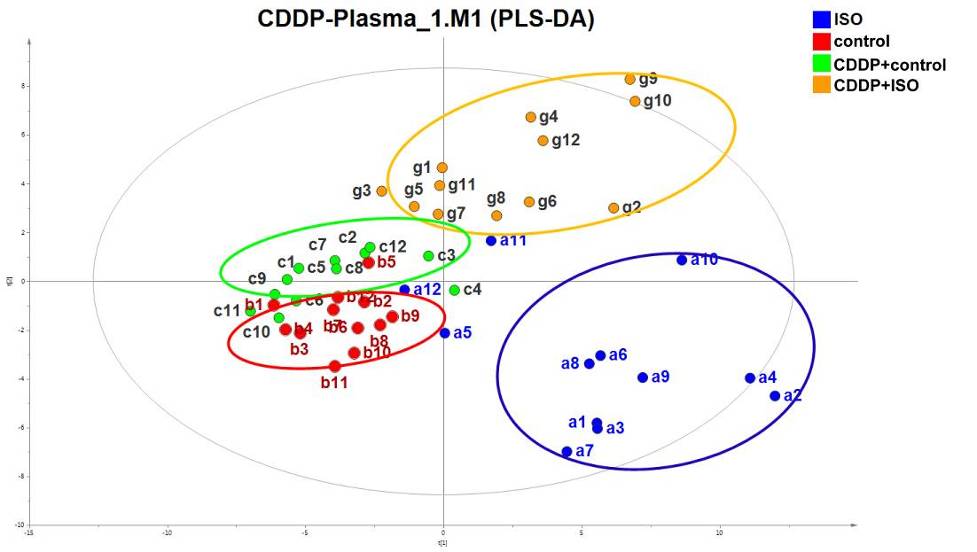


## Figure S2. The PLS-DA scores plots based on the metabolites in plasma of the ischemia myocardial model rats and the rats pre-treated with CDDP.

a: ISO; b: control; c: CDDP+ control; g: CDDP+ISO.

**Figure S3. OPLS analysis revealed the different metabolic pattern and the discriminant metabolites induced by ISO and CDDP based on metabolites in plasma.**

A, OPLS between ISO-induced model and the normal control; B, OPLS between CDDP pretreatment in ISO-induced model and ISO-induced model; C, S-plot analysis of metabolites differentiating ISO-induced model and the normal control; D, S-plot analysis of metabolites differentiating CDDP pretreatment in ISO-induced model and ISO-induced model.


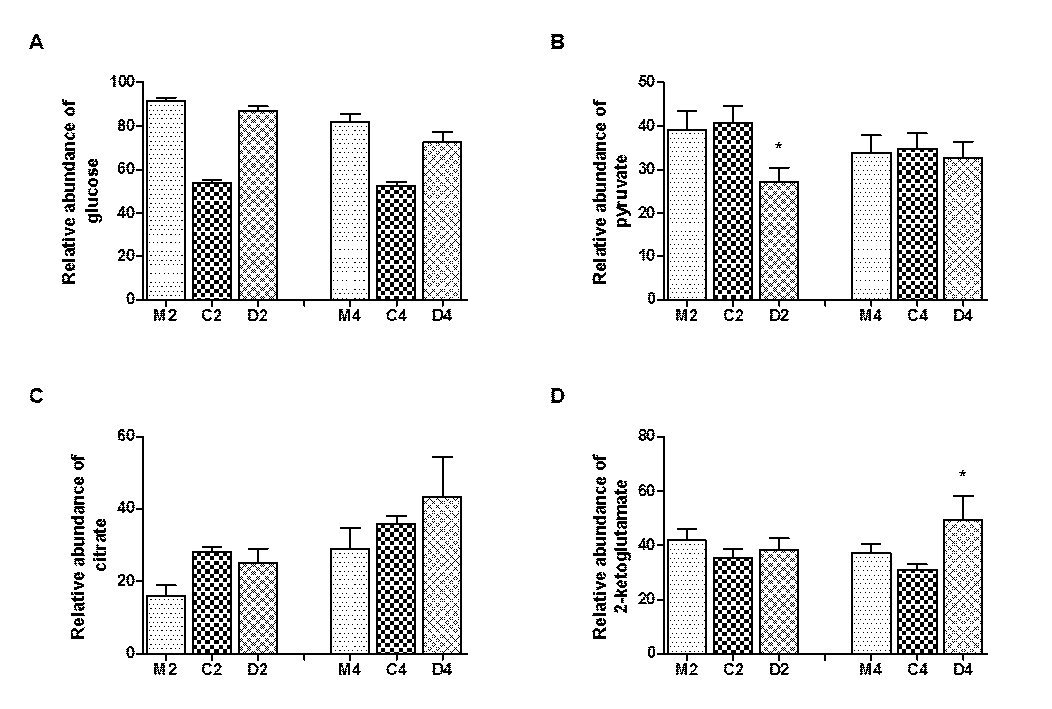


**Figure S4. The effect of ISO and CDDP on plasma metabolites involved in glycolysis and TCA.**

The elevated glucose indicated the insufficient usage of glucose induced by ISO. CDDP increased TCA turnover and hence the energy metabolism, that can be reflected by reduced pyruvate and increased citrate and 2-ketoglutarate at system level. *: p<0,05 vs the model (ANOVA). A, glucose; B, pyruvate; C, citrate; D, 2-ketoglutarate. M2: ISO 2 h; C2: control 2 h; D2: CDDP+ISO 2 h; M4: ISO 4 h; C4: normal 4 h; D4: CDDP+ISO 4 h.


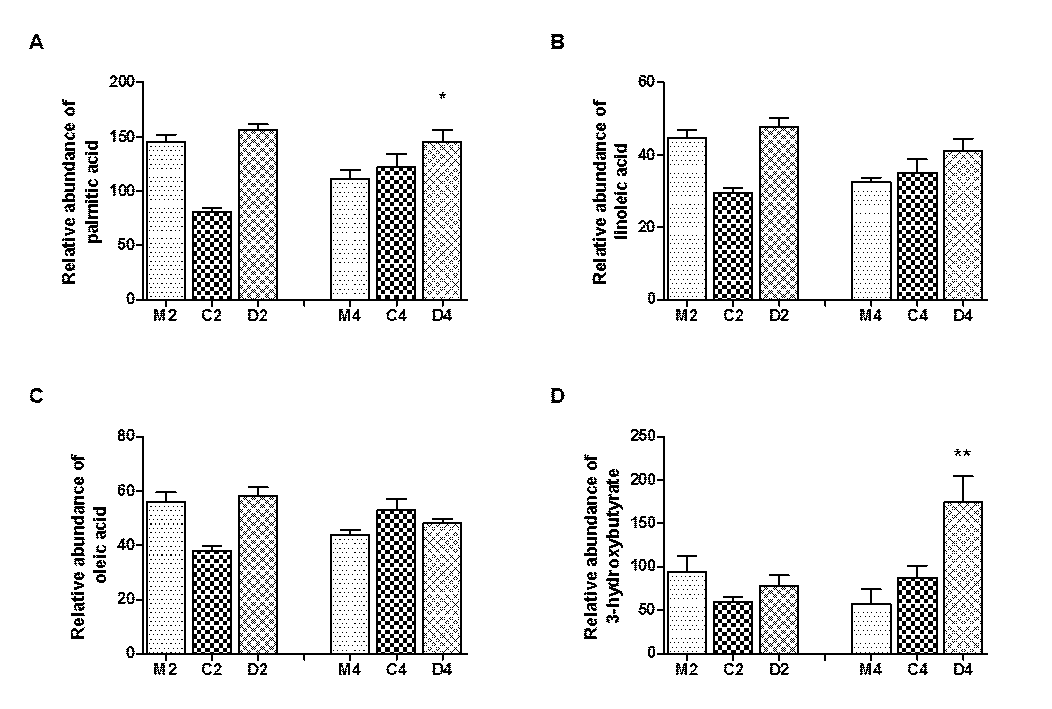


**Figure S5. The effect of ISO and CDDP on plasma metabolites involved in metabolism of free fatty acids.**

ISO induced elevation of fatty acids (palmitic acid, linoleic acid and oleic acid) and 3-hydroxybutyrate, indicating a decreased utilization of fatty acids for energy supply. Although CDDP showed marginal effect on levels of fatty acids, level of 3-hydroxybutyrate significantly increased at 4 hours, indicating an enhanced beta-oxidation of fatty acids. *, **: p<0.05, 0.01 vs the model (ANOVA). A, palmitic acid; B, linoleic acid; C, oleic acid; D, 3-hydroxybutyrate. M2: ISO 2 h; C2: control 2 h; D2: CDDP+ISO 2 h; M4: ISO 4 h; C4: control 4 h; D4: CDDP+ISO 4 h.


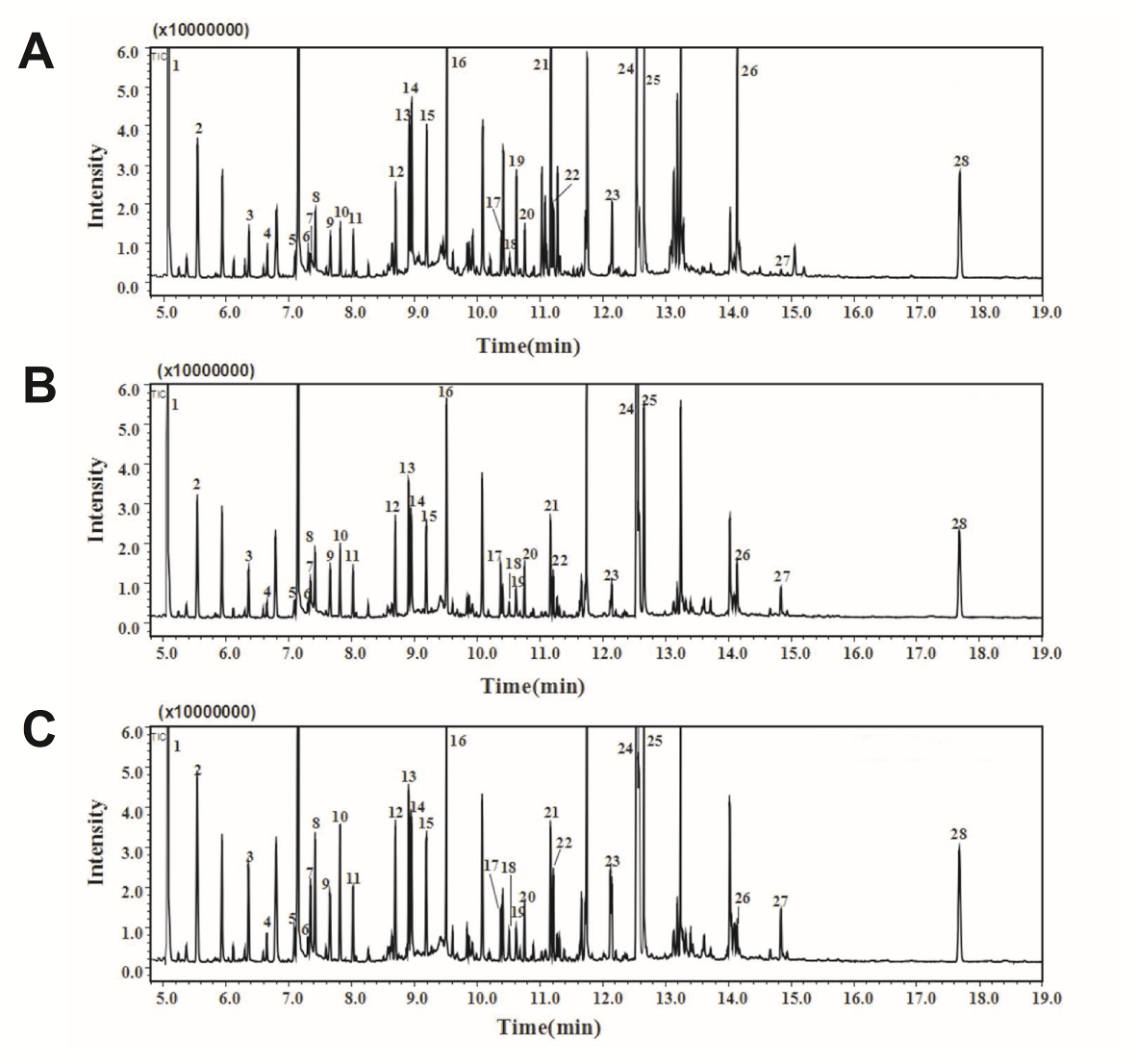


Figure S6. Typical GC/MS chromatograms of heart tissue extracts.

A, control group; B, ISO group; C, CDDP+ISO group. Visual inspection of the chromatograms revealed obvious differences between the normal control, ISO-induced model and CDDP pretreatment in ISO-induced model. The molecules were identified as: 1, Lactate; 2, Alanine; 3, Urea; 4, Valine; 5, Isoleucine; 6, Leucine; 7, Proline; 8, Glycine; 9, 2 peaks ( Uracil, Fumarate); 10, Serine; 11, Threonine; 12, Malate; 13, Aspartate; 14, Pyroglutamate; 15, Creatinine; 16, Glutamate; 17, Glycero-2-phosphate; 18, O-Phosphorylethanolamine; 19，Hypoxanthine; 20, Citrate; 21, Glucose; 22, Lysine; 23, myo-Inositol; 24, Oleic acid; 25, Stearic acid; 26, Inosine; 27, 1-Monooleoylglycerol; 28, Cholesterol.


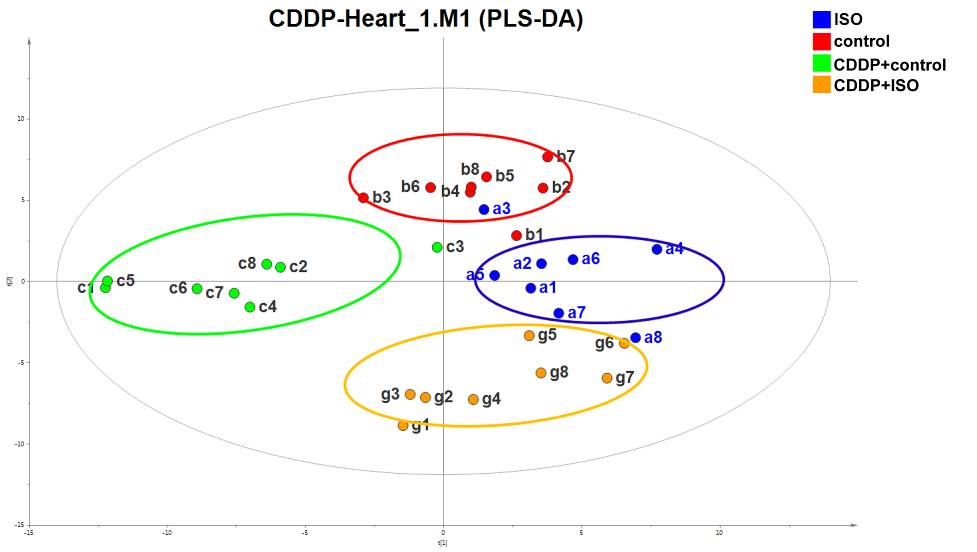


## Figure S7. The PLS-DA scores plots based on the metabolites in heart tissue of the ischemia myocardial model rats and the rats pre-treated with CDDP.

a: ISO-induced model; b: Normal control; c: CDDP pretreatment in normal control; g: CDDP pretreatment in ISO-induced model.

Figure S8. Multivariate statistic analysis of the tissue metabolites from the ischemia myocardial model rats and the rats administrated with CDDP for 2 h.

The three components PLS-DA model showed that ISO perturbed metabolic pattern of tissue metabolome, while CDDP withheld the tendency induced by ISO after administration with CDDP for 2 h. The PLS-DA parameters: PC1, R^2^X=0.351, R^2^Y=0.493, Q^2^=0.478; PC2, R^2^X=0.583, R^2^Y=0.680, Q^2^=0.552; PC3, R^2^X=0.654 R^2^Y=0.918, Q^2^=0.779; Pemutation test, R^2^=0.253, Q^2^=-0.193. A, PLS-DA model, PC1-PC2; B, PLS-DA model, PC1-PC3; C, PLS-DA model, PC2-PC3; D, OPLS model.

Figure S9. Multivariate statistic analysis of the tissue metabolites from the ischemia myocardial model rats and the rats administrated with CDDP for 4 h.

The three components PLS-DA model showed that ISO perturbed metabolic pattern of tissue metabolome, while CDDP withheld the tendency induced by ISO after administration with CDDP for 4 h. The PLS-DA parameters: PC1, R^2^X=0.339, R^2^Y=0.472, Q^2^=0.460; PC2, R^2^X=0.550, R^2^Y=0.745, Q^2^=0.689; PC3, R^2^X=0.603, R^2^Y=0.897, Q^2^=0.783; Pemutation test, R^2^=0.134, Q^2^=-0.285. A, PLS-DA model, PC1-PC2; B, PLS-DA model, PC1-PC3; C, PLS-DA model, PC2-PC3; D, OPLS model.

Figure S10. OPLS analysis revealed the different metabolic pattern and the discriminant metabolites induced by ISO and CDDP based on metabolites in heart tissue.

A, OPLS between ISO-induced model and the normal control; B, OPLS between CDDP pretreatment in ISO-induced model and ISO-induced model; C, S-plot analysis of metabolites differentiating ISO-induced model and the normal control; D, S-plot analysis of metabolites differentiating CDDP pretreatment in ISO-induced model and ISO-induced model.


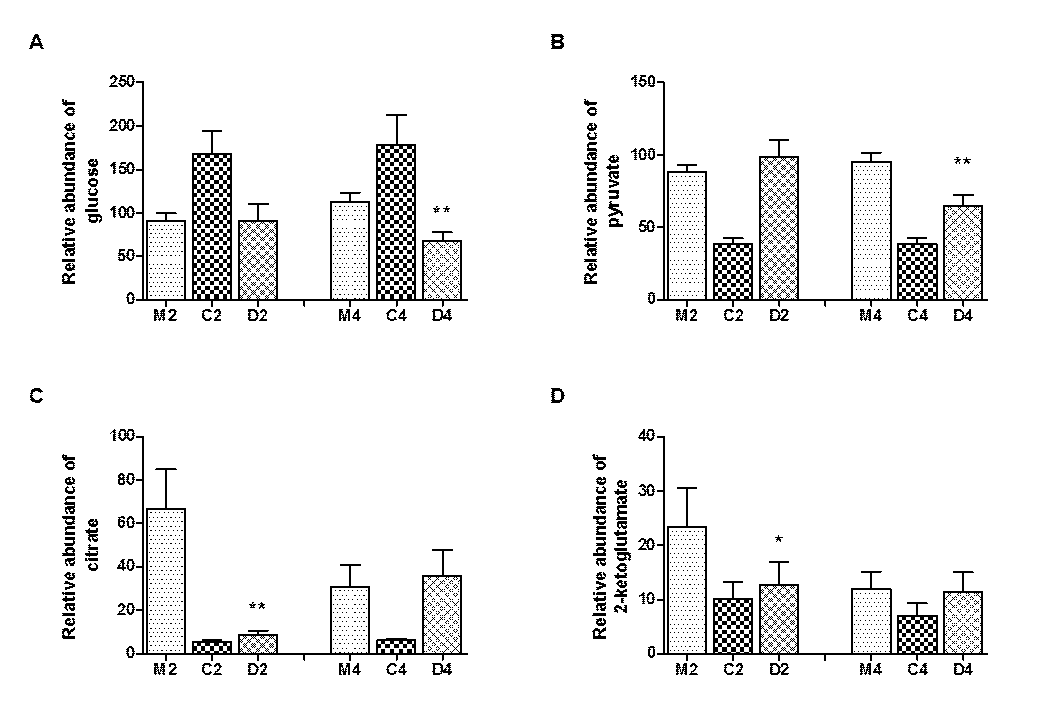


Figure S11. The effect of ISO and CDDP on tissue metabolites involved in glycolysis and TCAs.

The decreased glucose in tissue indicated an insufficient blood supply induced by ISO. The significantly elevated TCA intermediates (citrate and 2-ketoglutarate) suggested the inhibited TCA cycle and hence the decreased energy metabolism. CDDP greatly lowered down levels of citrate and 2-ketoglutarate, suggesting an increased TCA and energy supply, which was supported by the more generation of ATP. *, **: p<0.05, 0.01 vs the model (ANOVA). M2: ISO 2 h; C2: control 2 h; D2: CDDP+ISO 2 h; M4: ISO 4 h; C4: control 4 h; D4: CDDP+ISO 4 h.


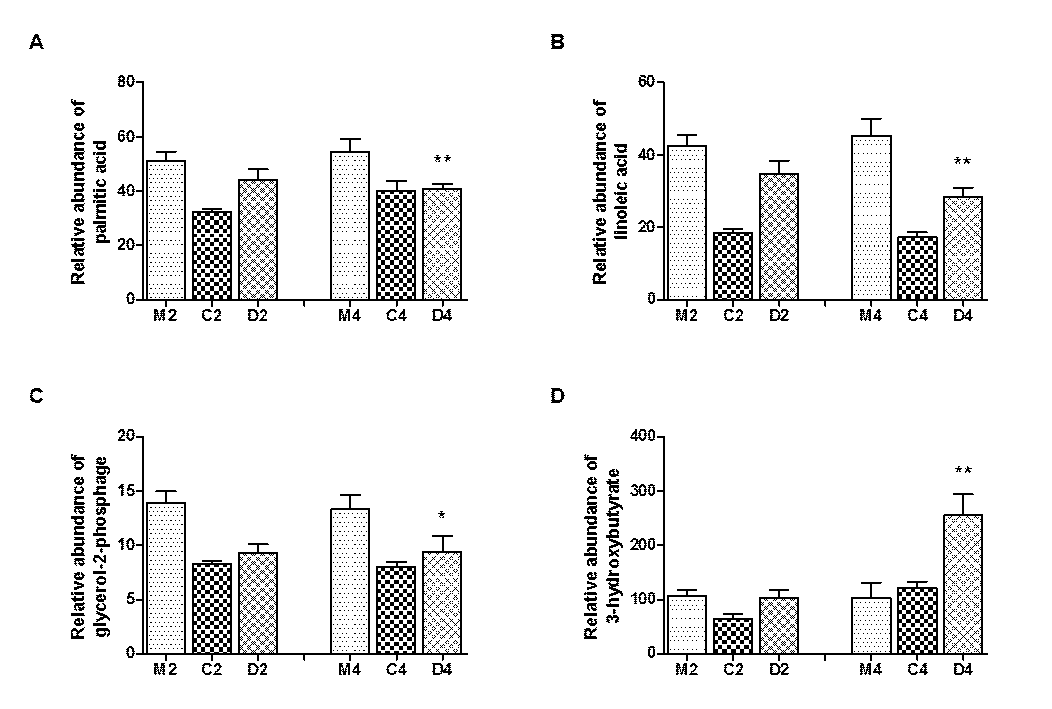


**Figure S12. The effect of ISO and CDDP on tissue metabolites involved in metabolism of free fatty acids.** ISO induced elevation of citrate (2 h) and lipids (glycerol-2-phosphagte, palmitic acid, linoleic acid and oleic acid) and a reduction of 3-hydroxybutyrate(4h), indicating a decreased utilization of fatty acids for energy supply. Although CDDP showed less effect on levels of fatty acids, level of 3-hydroxybutyrate significantly increased at 4 hours, indicating an enhanced beta-oxidation of fatty acids. *, **: p<0.05, 0.01 vs the model (ANOVA). M2: ISO 2 h; C2: control 2 h; D2: CDDP+ISO 2 h; M4: ISO 4 h; C4: control 4 h; D4: CDDP+ISO 4 h.
